# Comprehensive characterization of volatile terpenoids and terpene synthases in *Lanxangia tsaoko*

**DOI:** 10.1186/s43897-024-00140-0

**Published:** 2025-04-03

**Authors:** Shanshan Chen, Mofan Zhang, Shuo Ding, Zhichao Xu, Sifan Wang, Xiangxiao Meng, Shilin Chen, Ranran Gao, Wei Sun

**Affiliations:** 1 State Key Laboratory for Quality Ensurance and Sustainable Use of Dao-di Herbs, Institute of Chinese Materia Medica, China Academy of Chinese Medical Sciences, Beijing, 100700 China; 2 College of Life Science, Northeast Forestry University, Harbin, 150040 China; 3 Institute of Herbgenomics, Chengdu University of Traditional Chinese Medicine, Chengdu, 611137 China; 4 Artemisinin Research Center, Institute of Chinese Materia Medica, China Academy of Chinese Medical Sciences, Beijing, 100700 China

**Keywords:** Volatile organic compounds, Terpene synthase, *Lanxangia tsaoko*, Monoterpenoids, Sesquiterpenoids

## Abstract

*Lanxangia tsaoko* is widely utilized in human cuisine as a popular flavoring agent due to its distinctive aroma. It also has a long history of use in traditional Chinese medicine. The edible and medicinal properties of *L. tsaoko* are primarily attributed to its diverse array of volatile metabolites. Previous research has mainly focused on classifying the constituents and their pharmacological activities in *L. tsaoko*, leaving gaps in comprehensive identification and elucidation of the biosynthetic mechanisms of these metabolites. In this study, we employed a multi-omics approach and functional characterization to investigate the biosynthesis of volatile terpenoids in *L. tsaoko*. The results demonstrated that terpenoids constituted the highest proportion of volatile compounds in *L. tsaoko*. Additionally, 42 terpene synthase (TPS) coding genes were identified through genome-wide analysis. Functional characterization revealed that eight LtTPSs effectively catalyzed geranyl pyrophosphate to produce monoterpenoids, while four LtTPSs converted farnesyl pyrophosphate to generate sesquiterpenoids. Genome-wide and single-gene duplication events contributed to functional diversification among LtTPSs with high identity, promoting the diversity of terpenoids. These findings provide a foundation for understanding the biosynthesis of volatile terpenoids in *L. tsaoko*, enhance the current knowledge of TPS, and contribute to the broader understanding of the biochemical diversity of terpenoids in plants.

**Supplementary Information:**

The online version contains supplementary material available at 10.1186/s43897-024-00140-0.

## Core

This investigation employed a multidisciplinary approach, integrating transcriptomics, metabolic profiling, and functional characterization of terpene synthase (TPS)-encoding genes, to examine the volatile terpenoids in *L. tsaoko*. Metabolomic analysis revealed terpenoids as the predominant volatile compounds. Functional identification demonstrated that 10 LtTPSs belonging to the TPS-a/b subfamily could catalyze geranyl pyrophosphate (GPP) or farnesyl pyrophosphate (FPP) to produce mono/sesqui-terpenoids. These findings offer valuable insights into the molecular mechanisms driving the diversity and abundance of volatile terpenoids, which are essential for advancing research in biotechnology and natural product development.

## Gene & accession numbers

Information for the genes discussed in this article is available in the GenBank database, maintained by the National Center for Biotechnology Information (NCBI), under accession numbers PQ448317-PQ448325.

## Introduction

*Lanxangia tsaoko*, a perennial herbaceous plant of the Zingiberaceae family, is widely distributed in southeastern China, parts of Vietnam, and northern Laos (Wei et al. 2019). Its fruit, known locally as "Cao Guo" in Chinese, is utilized both as a significant food additive and spice for masking odors and enhancing flavor, and as a traditional Chinese medicine effective in treating various disorders. The distinctive aroma of *L. tsaoko* is primarily attributed to its rich content of volatile organic compounds (VOCs), including monoterpenoids, sesquiterpenoids, and fatty acids, which have garnered attention due to their diverse biological activities. For instance, monoterpenoids derived from *L. tsaoko* demonstrate anti-inflammatory (Liang et al. 2024) and anti-*Trichomonas vaginalis* activities (Dai et al. 2016). Given its high edible and medicinal value, *L. tsaoko* has been the subject of extensive research in recent years. However, these studies on the active ingredients of *L. tsaoko* have primarily focused on the identification and classification of constituents (Liu et al. 2018; He et al. 2020; Shi et al. 2021). Further investigation is required into the biosynthetic pathways and regulatory mechanisms governing VOCs in *L. tsaoko*. In plant tissues, VOCs are synthesized to serve important biological functions, including resistance against pathogenic bacteria, parasitic organisms, and herbivores (Rawat et al. 2024). Therefore, research on VOCs in *L. tsaoko* could enhance our understanding of its aroma formation mechanisms and provide insights into its growth, development, and ecological adaptation.

VOCs are classified into terpenoids, benzene aromatics, and fatty acid derivatives based on their origin, with terpenoids forming the predominant class (Holopainen and Gershenzon 2010). Terpenoids, also referred to as isoprenoids, represent the largest and most structurally diverse group of natural products in plants (Ahmad et al. 2024). These compounds play crucial roles in chemical ecology, influencing interactions with herbivores, pathogens, and symbiotic organisms (Tholl 2015; Nagegowda and Gupta 2020; Wei et al. 2023; Cao et al. 2024; Jun Yang and Wang, 2024). For example, monoterpenoids and sesquiterpenoids function as indirect defense mechanisms by deterring herbivores or attracting their natural predators (Gershenzon and Dudareva 2007). Furthermore, terpenoids contribute to the production of essential oils and resins, which protect plants against microbial infections and environmental stressors (Pichersky and Raguso 2018; Yadav et al. 2024). Volatile terpenoids, primarily comprising isoprene (C5), monoterpenes (C10), and sesquiterpenes (C15), constitute the largest class of plant volatile compounds (Dudareva et al. 2006). Notably, the more volatile monoterpenoids and sesquiterpenoids play diverse roles in plant growth, development, and ecological fitness (Chen et al. 2011). These functions encompass both direct and indirect forms of defense, the enhancement of flavor and aroma in herbs and fruits, and antioxidant activities (Allen et al. 2019). Due to their significant medicinal value and relatively low abundance in native species, research into the biosynthesis pathways and metabolic engineering of plant-specialized sesquiterpenoids and monoterpenoids has gained increasing attention (Liu et al. 2024).

The classical cytosolic mevalonate (MVA) pathway synthesizes isopentenyl diphosphate (IPP) from acetyl-CoA (Newman and Chappell 1999), playing a vital role in sesquiterpenoids and sterol production (Nes 2011). Conversely, the plastidial 2-C-methyl-D-erythritol-4-phosphate (MEP) pathway generates IPP and dimethylallyl diphosphate (DMAPP) from pyruvate (Wang et al. 2024), and is fundamental for synthesizing monoterpenoids, diterpenoids, and carotenoids (Lichtenthaler 1999). IPP and DMAPP originate from two distinct biosynthetic pathways localized in different subcellular compartments (Kubeczka 2020; Wu et al. 2024). In some plants, DMAPP formed in plastids serves as a precursor for isoprene synthesis catalyzed by isoprene synthase (ISPS). All terpenoids are synthesized from these universal five-carbon precursors. IPP and DMAPP undergo further condensation by prenyl diphosphate synthases within their respective compartments (Zhao et al. 2023b; Yang et al. 2024). These prenyl diphosphate intermediates then serve as substrates for a diverse array of terpene synthase (TPS) (Wang et al. 2023), encoded by large *TPS* gene families comprising eight subfamilies. Historically, TPSs were classified into two types based on their structure and catalytic mechanism: type I and type II (Chen et al. 2011; Gao et al. 2012). Recent classifications based on sequence similarities and functional features have expanded this categorization into eight subfamilies: TPS-a, TPS-b, TPS-c, TPS-d (found in gymnosperms only), TPS-e/f, TPS-g, and TPS-h (in *Selaginella* spp.). Among these, TPS-c belongs to Type II TPS while others belong to Type I (Wang et al. 2019b). Type I TPSs are characterized by an aspartic acid-rich DDXXD motif (*α*-domain) in their C-terminal domain, which binds metal cofactors (Mg^2^⁺ or Mn^2^⁺). These cofactors are essential for interacting with isoprenyl diphosphate substrates and facilitating substrate cation formation (Christianson 2017)*.* In contrast, type II terpene synthases possess a DXDD motif in the β-domain near the N-terminal (Zerbe and Bohlmann 2015). The catalytic activity of TPS enzymes results in the formation of an extensive range of terpenoid compounds, including mono-, sesqui-, and diterpenes, as well as other specialized metabolites crucial for plant adaptation and defense mechanisms (Alicandri et al. 2020).

In recent years, *L. tsaoko* has attracted significant attention due to its high concentration of bioactive constituents, which show potential for application as food additives and in pharmaceutical development. Among these, terpenoids constitute a major class of compounds in *L. tsaoko*. Nevertheless, the biochemical and molecular basis of terpenoid biosynthesis remains unclear. Therefore, this study aimed to conduct a comprehensive analysis and investigation of volatile compounds in *L. tsaoko*, examining their compositional characteristics and biosynthetic pathways. This research endeavored to provide a scientific foundation for the industrial utilization and functional assessment of *L. tsaoko*. Based on structural domain searches and functional annotations, 42 *LtTPSs* were identified in this study, with 10 candidate *LtTPSs* functionally characterized to elucidate the genetic basis of volatile terpenoid enrichment. The identification of terpenoids and the *TPS* gene family in *L. tsaoko* provides crucial target genes for in-depth investigation of volatile oil biosynthesis regulation in this species. In conclusion, this study offers novel insights for investigating the biosynthesis mechanisms of diverse volatile terpenoids in Zingiberaceae species, while also providing genetic resources for the improvement of this medicinal plant and the evolutionary analysis of the ginger family.

## Results

### Volatile metabolite profiles from *L. tsaoko* using GC–MS

**Fig. 1:**
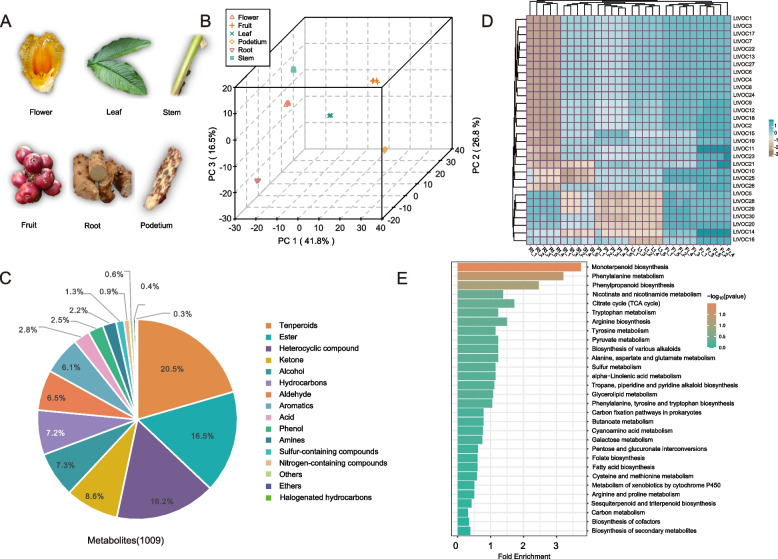
Six tissues metabolite profiles from *L. tsaoko*. **A** Principal Component Analysis (PCA) of metabolites detected in *L. tsaoko*. The X-axis and Y-axis indicate PC1 and PC2, respectively. **B** The distribution of the metabolites. **C** The relative content heatmap (row scale) of the top 30 volatile compounds in six different tissues. R: root, S: stem, L: leaf, Fr: fruit, Fl: flower, P: podetium. **D** The top 30 KEGG enrichment pathways. The X-axis position and size of the bar indicate the enrichment degree of the DAMs. The color of bars represents the *P-value*

We conducted gas chromatography-mass spectrometry (GC–MS) analysis to evaluate volatile compounds in six distinct tissues: root, stem, flower, leaf, fruit, and podetium (Fig. 1A). Principal Component Analysis (PCA) was employed to assess the metabolic profiles of these samples, revealing consistent patterns within the same group, thus demonstrating the reliability and reproducibility of our detection results. The separation trend between different tissues was evident, indicating significant metabolic differences among these tissues (Fig. 1B). A total of 1009 volatile compounds were detected (Table S1) and categorized into 15 classes. Notably, terpenoids (20.5%) emerged as the predominant class, followed by esters (16.5%), heterocyclic compounds (16.2%), and ketones (8.6%) (Fig. 1C). Furthermore, the Orthogonal Partial Least Squares Discriminant Analysis (OPLS-DA) plot demonstrated significant metabolic alterations among different tissues, with R2X, R2Y, and Q^2^ values of 0.821, 0.992, and 0.996, respectively (Fig. S1, Table S2). The Q2 values among the six tissues ranged from 0.9 to 1, confirming the robustness and efficiency of the results and methods. This approach facilitated the identification of differentially accumulated metabolites (DAMs) using Variables important in the projection (VIP) analysis.

**Fig. 2:**
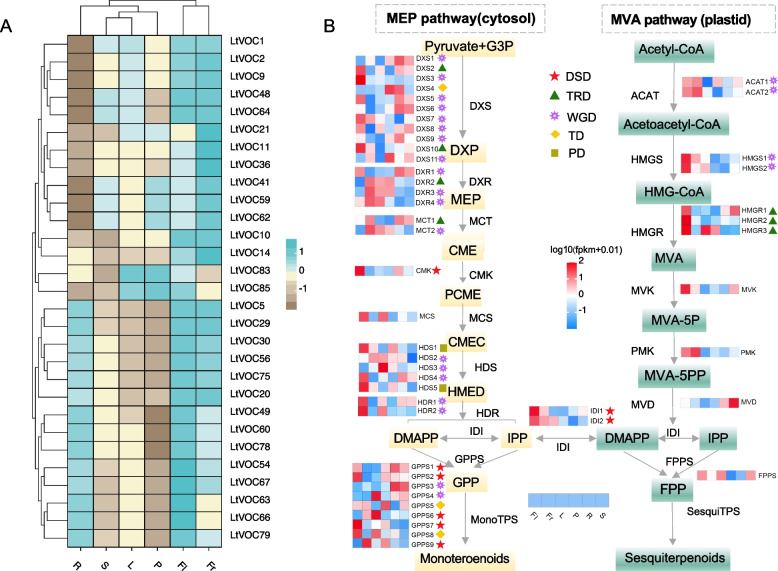
Volatile terpenoids and terpenoid precursor biosynthesis pathways in *L. tsaoko.* **A** Heatmap depicting the distribution of volatile terpenoid content across six distinct tissues (R: root, S: stem, L: leaf, Fr: fruit, Fl: flower, P: podetium) of *L. tsaoko.* **B** Tissue-specific expression profiles of genes involved in terpenoid precursor biosynthesis (heatmap, row scale). The heatmap illustrates tissue-specific expression patterns of genes participating in the biosynthesis pathways of terpenoid precursors. Different shapes in the lower panel indicate different duplication event origins of the upstream genes

To further analyze the distribution of VOCs across different tissues, we quantified the content of all volatile compounds. The results revealed the highest concentrations in fruits, followed by flowers. This distribution may be closely associated with the culinary applications of *L. tsaoko* fruits and the floral scents (Fig. S2). A barplot of the 30 most abundant compounds demonstrated the prevalence of terpenoids, indicating their significant contribution to the overall volatile compound composition. Notably, terpenoids were more abundant in flowers and fruits compared to the other four tissues. For instance, α-pinene and β-*cis*-ocimene were most concentrated in flowers, while citral, o-cymene, pseudolimonene, and *trans*-anethole were more prevalent in fruits. Eucalyptol was enriched across five tissues, excluding the roots (Fig. S3). A heatmap constructed from this data set further confirmed that the top 30 volatile compounds were primarily detected in flowers and fruits (Fig. 1D). KEGG enrichment analysis of the differential metabolites indicated significant enrichment in monoterpenoid biosynthesis (Fig. 1E). These findings aligned closely with the utilization of *L. tsaoko* fruits as a spice and in traditional Chinese medicine. The identification of volatile compounds in *L. tsaoko* revealed terpenoids as the primary active components. Subsequent analysis of volatile terpenoids across six distinct tissues, focusing on the 30 most abundant terpenoids, demonstrated that flowers and fruits possessed the richest repertoire, with most monoterpenoids and sesquiterpenoids highly concentrated in these tissues (Fig. 2A).

### Identification of upstream genes related to terpenoid biosynthesis

The expression patterns of upstream synthases involved in the MEP and MVA pathways play a crucial role in regulating the biosynthesis of flavor terpenoids (Qiao et al. 2021). We initially identified key genes involved in terpenoid backbone biosynthesis, revealing multiple copies of most terpenoid pathway genes. Notably, *DXS* has eleven copies, *DXR* has four copies, and *HDS* has five copies. To investigate the origin of these multiple copies, we identified various gene duplication modes, including whole genome duplication (WGD), tandem duplication (TD), proximal duplication (PD), transposed duplication (TRD), and dispersed duplication (DSD). Among these, TD, PD, TRD, and DSD were classified as single-gene duplication events (Fig. S4). The results indicated that WGD events significantly contributed to the multiplication of upstream genes involved in specific terpenoid biosynthesis (Fig. 2B). Transcriptomic analysis revealed distinctive expression patterns. *LtDXS2/3/5/7/8/10* exhibited the highest expression in flowers, while *LtDXS1/6/9* showed relatively higher expression in roots compared to the other eight *LtDXSs*. Three of the four *DXR* genes demonstrated peak expression in fruits. Among the five *HDS* genes, three (*HDS1*/*4*/*5*) showed elevated expression levels in flowers. Additionally, although the *MCT*, *CMK*, *MCS*, and *HDR* genes exhibited only single or double copies in the genome, they were considerably expressed in flowers or fruits. These transcriptomic data aligned with the metabolomic results, suggesting these genes' potential involvement in monoterpenoid biosynthesis. In the MVA pathway, despite the absence of multiple gene copies, most upstream genes were highly expressed in flowers and fruits. For instance, both *ACAT* and *HMGS* were present as two copies, primarily derived from WGD events. The three copies of *HMGR* were generated through TRD. *ACAT*, *HMGS1*, and *phosphomevalonate kinase* (*PMK*) exhibited high expression levels in both flowers and fruits. The single copy of *MVK* displayed elevated expression levels in flowers. These highly expressed upstream genes could have facilitated terpenoid accumulation in flowers and fruits. Metabolomic analyses further corroborated this finding by detecting high accumulation of key terpenoids in flowers and fruits (Fig. 2B). Furthermore, the correlation structure at a broader scale for the top 30 volatile terpenoids was presented (Fig. S5). Notable correlations include those between citral and *trans*-carvone oxide, as well as limonene and β-myrcene, suggesting a potential shared biosynthesis pathway.

### Phylogenetic analysis, structural and expression pattern of *LtTPSs*

**Fig. 3:**
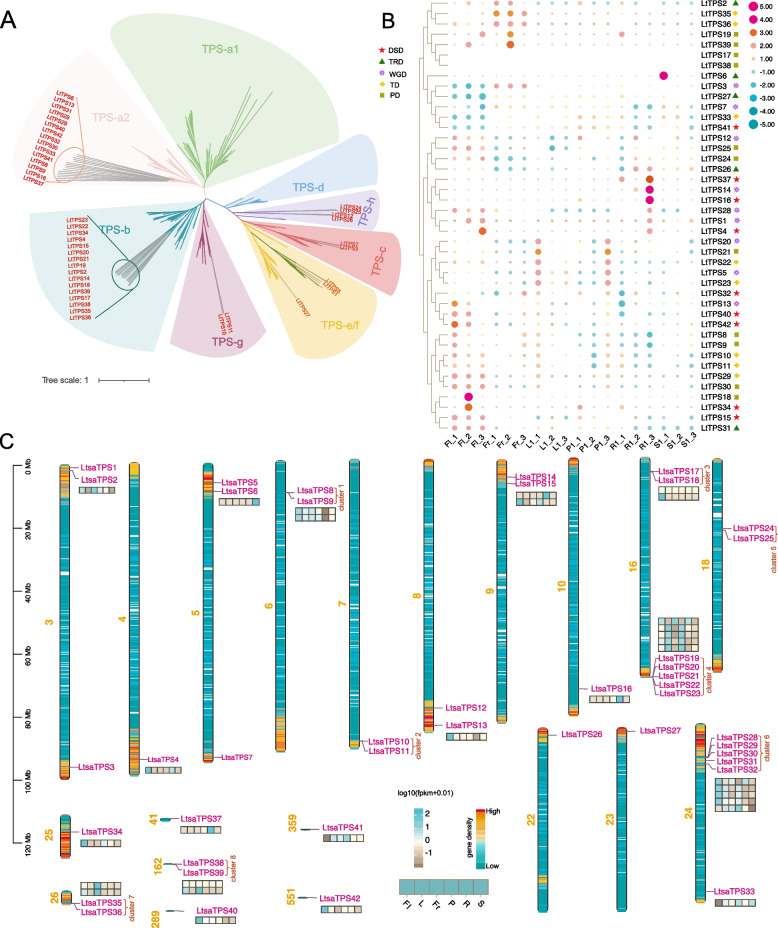
Analysis of *TPS* gene family in *L. tsaoko. ***A** Phylogenetic tree and subfamily classification of LtTPSs with TPSs from other species. The gene ID in red represents TPS in *L. tsaoko*. **B** Heatmap illustrating the expression of *LtTPSs* across various tissues (R: root, S: stem, L: leaf, Fr: fruit, Fl: flower, P: podetium) based on transcriptome data. The different shapes on the right side of the expression heatmap indicate different replication event origins of the TPS genes. **C** Schematic map representation of the genomic localization of *LtTPSs*

TPSs are responsible for the biosynthesis of diverse terpenoid compounds. A total of 42 putative *LtTPSs* were identified in the genome of *L. tsaoko* (Table 1). Based on their chromosomal location, the *LtTPSs* were designated *LtTPS1*-*LtTPS42*. The physicochemical properties were analyzed using the ProtParam online tool, including the open reading frame (ORF) length, molecular weight (MW), grand average of hydropathicity (GRAVY), and isoelectric point (pI). The ORF length varied from 294 amino acids (LtTPS11) to 1040 amino acids (LtTPS12), with MW ranging from 33,853.59 Da (LtTPS11) to 118,944.45 Da (LtTPS12). GRAVY values spanned from −0.18 (LtTPS5) to −0.52 (LtTPS2), and pI ranged between 4.9 (LtTPS23) and 6.39 (LtTPS9) (Table 1). Subsequently, a phylogenetic tree of 42 LtTPSs was constructed with 283 representative TPSs from seven other species. The 42 LtTPSs were classified into six subfamilies: TPS-a (15), TPS-b (16), TPS-c (2), TPS-e/f (3), TPS-g (2) and TPS-h (4) (Fig. 3A). Additionally, domain and motif analysis revealed that all LtTPSs of the TPS-a subfamily were characterized by a highly conserved DDXXD motif (α-domain) in their C-terminal domain, along with an additional NSE/DTE motif. The TPS-b subfamily in *L. tsaoko* primarily comprised monoterpene synthases (monoTPS) characterized by a conserved DDXXD motif and a relatively conserved RR(X)_8_W motif, essential for monoterpene cyclization (Fig S6, Tables S3 & S4).

**Table 1:** List of identified *TPS* genes from the *L. tsaoko* genome with their physiochemical properties

Sequence ID	Gene ID	TPS subfamily	Number of Amino Acid (AA)	Molecular Weight(Da)	Theoretical pI	GRAVY
LtTPS1	*Ltsa03G000072.t1*	e/f	770	88,555.26	6.13	−0.226
LtTPS2	*Ltsa03G000152.t1*	b	596	69,335.9	5.54	−0.52
LtTPS3	*Ltsa03G001905.t2*	c	797	90,036.84	5.22	−0.208
LtTPS4	*Ltsa04G002064.t1*	b	589	68,807.24	6.39	−0.458
LtTPS5	*Ltsa05G000463.t1*	e/f	763	87,303.87	6.24	−0.18
LtTPS6	*Ltsa05G000749.t1*	a2	524	61,155.4	5.67	−0.269
LtTPS7	*Ltsa05G001782.t1*	c	907	103,468.86	5.59	−0.295
LtTPS8	*Ltsa06G000216.t1*	a2	662	74,693.17	6.21	−0.327
LtTPS9	*Ltsa06G000220.t1*	a2	677	76,532.25	6.39	−0.332
LtTPS10	*Ltsa07G000966.t1*	g	647	74,381.09	5.8	−0.285
LtTPS11	*Ltsa07G000967.t1*	g	294	33,853.59	6.01	−0.338
LtTPS12	*Ltsa08G001103.t1*	h	1040	118,944.45	5.34	−0.38
LtTPS13	*Ltsa08G001687.t1*	a2	615	71,188.06	5.56	−0.351
LtTPS14	*Ltsa09G000535.t1*	b	499	58,200.34	5.14	−0.368
LtTPS15	*Ltsa09G000693.t1*	b	615	71,615.63	5.75	−0.438
LtTPS16	*Ltsa10G000989.t1*	a2	504	58,340.77	5.18	−0.254
LtTPS17	*Ltsa16G000164.t1*	b	576	66,935.14	5.62	−0.415
LtTPS18	*Ltsa16G000168.t1*	b	575	66,816.9	5.58	−0.412
LtTPS19	*Ltsa16G000964.t2*	b	578	65,785.89	5.26	−0.331
LtTPS20	*Ltsa16G000966.t1*	b	587	68,106.67	5.52	−0.423
LtTPS21	*Ltsa16G000968.t1*	b	587	68,101.63	5.55	−0.423
LtTPS22	*Ltsa16G000969.t1*	b	592	68,716.6	5.6	−0.312
LtTPS23	*Ltsa16G000970.t1*	b	508	59,354.76	4.9	−0.234
LtTPS24	*Ltsa18G000282.t1*	h	614	69,045.92	5.73	−0.195
LtTPS25	*Ltsa18G000286.t1*	h	605	68,490.7	6.39	−0.21
LtTPS26	*Ltsa22G000268.t1*	h	839	95,475.75	5.6	−0.383
LtTPS27	*Ltsa23G000159.t1*	e/f	681	78,381.62	5.56	−0.317
LtTPS28	*Ltsa24G001032.t1*	a2	546	64,171.36	5.19	−0.305
LtTPS29	*Ltsa24G001033.t1*	a2	533	62,518.54	5.22	−0.335
LtTPS30	*Ltsa24G001034.t1*	a2	560	65,495.89	5.43	−0.341
LtTPS31	*Ltsa24G001096.t1*	a2	546	64,175.43	5.36	−0.324
LtTPS32	*Ltsa24G001156.t1*	a2	550	64,303.44	5.17	−0.288
LtTPS33	*Ltsa24G001702.t1*	a2	485	56,356.54	5.41	−0.298
LtTPS34	*Ltsa25G000294.t1*	b	576	66,804.04	5	−0.311
LtTPS35	*Ltsa26G000334.t1*	b	645	74,803.31	5.7	−0.378
LtTPS36	*Ltsa26G000335.t1*	b	576	67,082.11	5.2	−0.424
LtTPS37	*Ltsa41G000001.t1*	a2	541	62,472.07	5.11	−0.366
LtTPS38	*Ltsa162G000003.t1*	b	585	68,025.58	5.76	−0.394
LtTPS39	*Ltsa162G000006.t1*	b	475	55,085.65	5.56	−0.388
LtTPS40	*Ltsa289G000001.t1*	a2	512	59,970.71	5.15	−0.268
LtTPS41	*Ltsa359G000001.t1*	a2	579	66,582.23	6.04	−0.289
LtTPS42	*Ltsa551G000001.t1*	a2	549	64,102.36	5.27	−0.294

Among 42 LtTPSs, the TPS-b (16) subfamily members were significantly expanded in *L. tsaoko* compared to other representative plants, while the TPS-a (15) subfamily also exhibited multiple gene copies (Table S5). Gene duplication event analysis revealed that DSD and PD, each involving 9 TPS-a/b homologs, primarily influenced TPS-a/b gene expansion, potentially contributing to the abundance and diversity of volatile terpenoids (Fig. 3B&C). As TPS-a and TPS-b subfamilies predominantly function as monoterpene synthases and sesquiterpene synthases (sesTPS), this suggests that these two subfamilies likely contribute to the substantial production of volatile monoterpenoids and sesquiterpenoids in *L. tsaoko*. Additionally, the expression patterns of *LtTPSs* were compared across six different tissues (Fig. 3B). Among the TPS-a or TPS-b members, *LtTPS8* and *LtTPS9*, *LtTPS13*, *LtTPS15*, *LtTPS29*, *LtTPS30*, *LtTPS31*, and *LtTPS42* demonstrated predominant expression in flowers (Fig. 3C, Table S6), indicating that these genes may play a critical role in the biosynthesis of volatile terpenoids in *L. tsaoko* flowers.

The 42 *LtTPSs* identified in this study were distributed across nine chromosomes and several unanchored contigs. Notably, 22 of these genes (52.4%) were located within gene clusters. Specifically, seven gene clusters, labeled as TPS-a and TPS-b, ranged in size from two to five genes each. Cluster 6, which contained *LtTPS28*, *LtTPS29*, *LtTPS30* and *LtTPS31*, were highly expressed in flowers, underscoring the significance of whole genome and single-gene duplication in floral terpenoid biosynthesis. Similarly, *LtTPS35* and *LtTPS36*, predominantly expressed in fruits, were tandemly duplicated genes clustered together, further emphasizing the crucial role of single-gene duplication in terpenoid production in both fruits and flowers.

### Functional characterization of *LtTPSs*

**Fig. 4:**
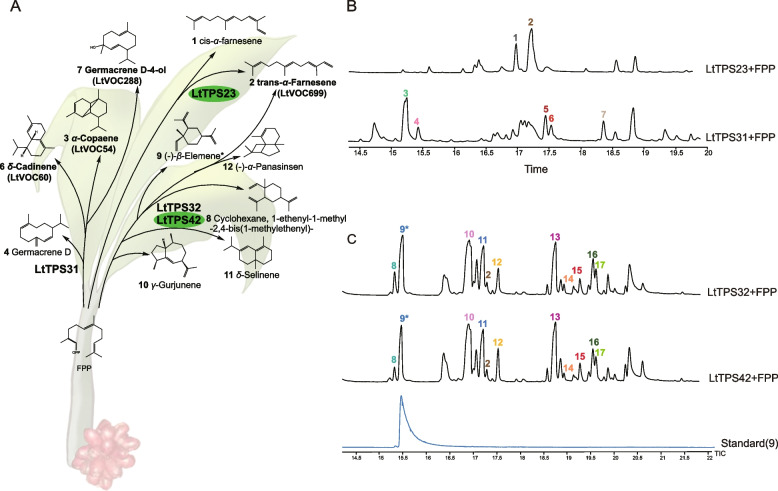
Functional analysis of LtTPS using FPP as the terpenoid precursor. **A** The sesquiterpenoid products of LtTPS23, LtTPS31, LtTPS32, and LtTPS42. Compounds in bold indicate metabolites identified in *L. tsaoko*, while those marked with an asterisk (*) correspond to the reference standards. Unannotated compounds are based on matches from the NIST standard reference database, and green-colored genes represent bifunctional genes. **B** The total ion current chromatogram (TIC) of the products from LtTPS23 and LtTPS31 with FPP as the substrate. **C** The TIC of the products from LtTPS32 and LtTPS42 with FPP as the substrate

**Fig. 5:**
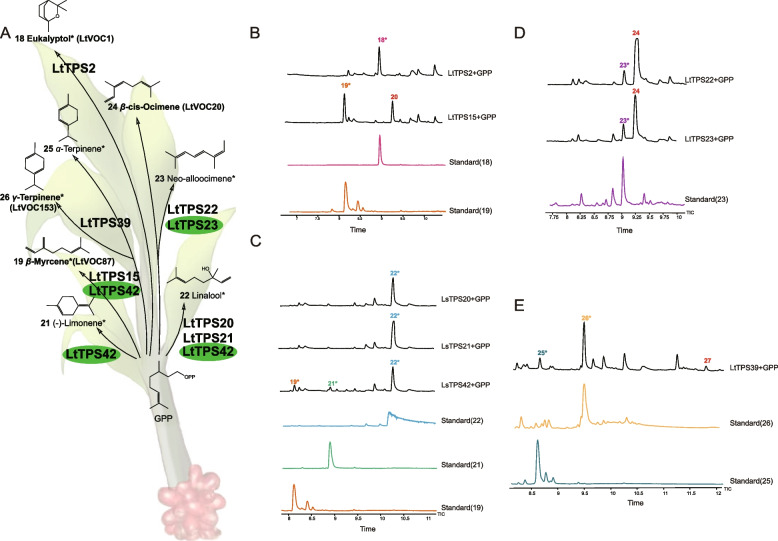
Functional analysis of LtTPS using GPP as the terpenoid precursor. **A** The sesquiterpenoid products of 8 LtTPSs. Bolded compounds indicate metabolites found in *L. tsaoko*. Compounds marked with an asterisk (*) represent those that correspond to the reference standards, while the green colored genes represent bifunctional genes. The unlabeled compounds correspond to the NIST standard reference database results. **B** The TIC of the product from LtTPS2 and LtTPS15 with GPP as the substrate. **C** The TIC of the product from LtTPS20, LtTPS21 and LtTPS42 with GPP. **D** The TIC of the product from LtTPS22 and LtTPS23 with GPP. **E** The TIC of the product from LtTPS39 with GPP

To further investigate the members of LtTPSs involved in terpenoid biosynthesis, 18 *LtTPSs*, including 7 *TPS-a* and 11 *TPS-b* subfamily members, were successfully cloned. Engineered strains producing farnesyl pyrophosphate (FPP) or geranyl pyrophosphate (GPP) were utilized to verify the function of candidate *LtTPSs*. The catalytic products from the 18 LtTPS enzymes using FPP or GPP as substrates were analyzed (Table S7). In comparison to the negative control (Fig S7), GC–MS detection revealed that four LtTPS enzymes (LtTPS23, LtTPS31, LtTPS32, and LtTPS42) exhibited sesquiterpene synthase activity, while eight LtTPS enzymes (LtTPS2, LtTPS15, LtTPS20, LtTPS21, LtTPS22, LtTPS23, LtTPS39, and LtTPS42) were found to convert GPP into monoterpenoids. Notably, LtTPS23 and LtTPS42 demonstrated bifunctional activity, underscoring their versatility in terpene synthesis (Figs. 4 and 5).

LtTPS23 converted FPP to *cis*-α-farnesene (1) and *trans*-α-farnesene (2) (Fig. 4A&B, Fig. S8). LtTPS31 primarily utilized FPP to produce α-copaene (3), germacrene D (4), ð-cadinene (6), and germacrene D-4-ol (7) (Fig. 4A&B, Fig. S9). LtTPS32 and LtTPS42 were found to mainly yield (-)-β-elemene (9), γ-gurjunene (10), δ-selinene (11), unknown sesquiterpenoids (13), and 7 additional byproducts (Fig. 4A&C, Fig. S10, Fig S11). Moreover, extensive functional analyses revealed that LtTPS32 and LtTPS42 exhibited the highest product diversity, highlighting their potential as key enzymes in terpenoid biosynthesis (Fig. 4). This suggests that LtTPS32 and LtTPS42 may play a crucial role in the diversity of volatile sesquiterpenoids in *L. tsaoko*.

Among the 11 TPS-b family genes cloned, LtTPS2, LtTPS15, LtTPS20, LtTPS21, LtTPS22, LtTPS23, and LtTPS39 demonstrated the ability to catalyze GPP, while LtTPS42 from TPS-a also exhibited monoterpene synthase activity. Specifically, LtTPS2 catalyzed the conversion of GPP into eucalyptol (18). LtTPS15 and LtTPS42 catalyzed the conversion of GPP into β-myrcene (19), which predominantly accumulated in flowers and fruits (Table S1), along with another unidentified monoterpenoid (20) (Fig. 5A&B, Fig. S12). LtTPS20 and LtTPS21, sharing 98.976% identity, exhibited identical catalytic products, producing linalool (22) (Fig. 5A&C, Fig. S13). Both LtTPS22 and LtTPS23 catalyzed GPP to yield neo-alloocimene (23) and β-*cis*-ocimene (24), with β-*cis*-ocimene predominantly accumulating in flowers and fruits (Fig. 5A&D, Fig. S14, Table S1). LtTPS22 was predominantly expressed in flowers, aligning with the observation that β-*cis*-ocimene accumulated most significantly in flowers (Table S2). Additionally, the main product of LtTPS39, γ-terpinene (26), accumulated at higher levels in flowers (Fig. 5A&E, Fig. S14, Table S1). These catalytic results indicate diverse catalytic activities among the TPS-b family members, contributing to the complexity of terpenoid biosynthesis in *L. tsaoko*. The TPS-a subfamily member LtTPS42 and the TPS-b subfamily member LtTPS23 exhibited the ability to catalyze both GPP and FPP. When catalyzing GPP, LtTPS42 produced linalool (22) as its main product, similar to the outputs of LtTPS20 and LtTPS21 (Fig. 5A&C, Fig. S13).

LtTPS32 and LtTPS42 exhibited a remarkably high sequence similarity of 99.091%, with only five amino acid differences (Fig S15). Despite this close genetic relationship, LtTPS32 lacked the monoterpene synthase activity observed in LtTPS42, underscoring the latter's unique bifunctionality. A similar pattern was observed with LtTPS22 and LtTPS23. These tandemly duplicated genes displayed a high degree of similarity, with LtTPS22 containing an additional 85 amino acids in the N-terminal region and 28 amino acid substitutions compared to LtTPS23 (Fig S16). Notably, while LtTPS23 catalyzed FPP to produce multiple sesquiterpenoids, LtTPS22 was limited to catalyzing GPP. These enzymatic results demonstrate that even minor alterations in amino acid sequences can result in significant variations in catalytic activity among TPS family members, reflecting the complex evolution of terpenoid biosynthesis pathways.

## Discussion

*L. tsaoko* is a medicinally and economically significant spice widely distributed in south-west China. The distinctive aroma of *L. tsaoko* is primarily attributed to its abundance of volatile compounds, particularly monoterpenoids and sesquiterpenoids, which enhance its flavor and culinary value. While previous research has focused mainly on the extraction of volatile oils and identification of certain bioactive compounds from dried fruits (Yang et al. 2008; Feng et al. 2010), this study provides a deeper molecular understanding of flavor terpenoids. In the biosynthesis of flavor terpenoid compounds, the expression patterns of the upstream synthases involved in the MEP and MVA pathways and TPS genes play critical roles. Upstream genes, particularly those involved in the synthesis of precursor compounds such as IPP and DMAPP, directly influence the efficiency of terpenoids biosynthesis (Wang et al. 2019a). Previous studies have shown that DXS overexpression in *Litsea cubeba* (Zhao et al. 2023a) and DXR regulation in *Salvia miltiorrhiza* can significantly impact monoterpenoids and sesquiterpenoids production (Zhou et al. 2016). This suggests that adjusting DXR and DXS can greatly affect the amount and type of terpenoid metabolites. In our comprehensive annotation of all genes of *L. tsaoko* involved in the MVA and MEP pathways, we identified specific genes with high expression levels in flowers and fruits, which may suggest tissue-specific precursor accumulation. For example, in the MEP pathway, such as *DXS2*, *DXS3*, *DXS5*, *DXS7*, *DXS10*, *HDS1*, *HDS4*, *HDS5* and *DXR1*, which showed predominant expression in flowers and fruits, potentially indicating terpenoid precursors accumulation in these tissues. Although we lack direct evidence of precursor accumulation, the high expression of upstream genes, including *HDR* in flowers, indicates a potential for efficient terpenoid synthesis in these tissues. In the MVA pathway, genes such as *ACAT1*, *HMGS*, *HMGR*, *MVK*, *PKM*, and *FPPS* also exhibited specific expression patterns, implying a similar pattern of precursor accumulation. Furthermore, our metabolite profiling identified monoterpenoids and sesquiterpenoids among the top 30 compounds with the highest concentrations in the analyzed samples, indicating a strong correlation between the elevated expression of MVA and MEP pathway genes and the high levels of terpenoids. The integration of gene expression data with metabolite profiles highlights the crucial role of the MEP pathway in monoterpenoid biosynthesis and emphasizes the differential accumulation of terpenoid precursors across various plant tissues. Additionally, KEGG enrichment analysis of the differential metabolites revealed that these compounds were primarily associated with the monoterpene synthesis pathway.

TPS serves as the crucial enzyme determining the structural diversity and skeleton of terpenoids (Chen et al. 2011). Investigations into the TPS gene families of *W. villosa* (Yang et al. 2022), *Eucalyptus grandis* (Myburg et al. 2014), and *Cinnamomum kanehira* (Chaw et al. 2019) have revealed significant expansions within the *TPS-a* and *TPS-b* subfamilies, likely playing a pivotal role in the biosynthesis and diversity of volatile sesquiterpenoids and monoterpenoids. This study similarly observed a substantial expansion in the TPS gene family, particularly within the *TPS-b* subfamilies. Prior research has demonstrated that tandem duplication acts as a major evolutionary force driving the expansion of the TPS gene family across various plant species (Karunanithi and Zerbe 2019; Chen et al. 2020; Wang et al. 2021). The current analyses of enzymes involved in terpenoid biosynthesis pathways further suggest that this expansion, predominantly facilitated by WGD and single-gene duplication, may have contributed to the observed terpenoid diversity in *L. tsaoko*. Transcriptome analysis revealed that more than half of the *LtTPS* genes were predominantly expressed in flowers and fruits, aligning with the high concentration of volatile terpenoids in these tissues and the enzymatic function of the genes in question. Consequently, a detailed investigation into the expression regulation of these upstream genes and TPS genes not only aids in elucidating the biosynthetic pathways of terpenoid compounds but also provides a theoretical foundation for genetic engineering approaches aimed at enhancing the yield of specific terpenoid compounds.

Previous studies have indicated that the catalytic products of TPSs are closely related to their sequence similarity (Karunanithi and Zerbe 2019). For instance, in rice (*Oryza sativa*) and maize (*Zea mays*), homologous TPS genes contribute to the biosynthesis of sesquiterpenoids such as *trans*-*β*-farnesene, which functions as a signal molecule in plant defense against insect herbivores (Schnee et al. 2006). In tomato, LeTPS28 and LeTPS48, sharing 85% amino acid sequence identity, both utilize *E*, *E*-FPP substrate to produce elemol and also catalyze the formation of an unidentified major sesquiterpenoid from *Z*, *Z*-FPP. Similarly, LeTPS33 and LeTPS35, with 91.7% sequence identity, catalyze the production of guaia-1(10),11-diene from *E*, *E*-FPP (Zhou and Pichersky 2020). In *L. tsaoko*, we identified a TPS gene cluster, which contained two pairs of duplication genes: *LtTPS20* and *LtTPS21*(PD), as well as *LtTPS22* and *LtTPS23*(TD), exhibiting a high degree of sequence similarity, respectively. Both LtTPS20 and LtTPS21 displayed identical catalytic activity for GPP. Interestingly, LtTPS23 possessed dual functionality and could utilize both GPP and FPP as a substrate, while LtTPS22 was restricted to catalyzing GPP (Figs. 4A, E, and 5). Consequently, predicting the products of a specific TPS based on homology with known TPS sequences is generally unreliable (Alquézar et al. 2017). It has been suggested that the product specificity of mono/sesTPS is influenced more by specific critical regions rather than overall sequence similarity. For example,* β*-pinene synthases in lemon and *Satsuma mandarin* (Lücker et al. 2002; Shimada et al. 2004) exhibit 95% and 97% sequence identity, respectively, with the sabinene synthase isolated from rough lemon. However, due to a single amino acid substitution, the latter cannot predominantly produce *β*-pinene (Kohzaki et al. 2009). Additionally, dispersed duplication resulted in copies of *LtTPS32* and *LtTPS42*, while LtTPS32 and LtTPS42 exhibited the same catalytic activity with FPP. Furthermore, LtTPS42 could also catalyze GPP to produce a variety of monoterpenoids, whereas LtTPS32 was limited to FPP (Figs. 4B, D, and 5). Similarly, CsTPS5 and CsTPS31 in *Cannabis sativa* are terpene synthases that arose through a gene duplication event. Although both enzymes can utilize FPP and GPP as substrates to produce various sesquiterpenoids and monoterpenoids, they exhibit distinct expression patterns and produce different specific terpenoids. This functional divergence likely enhances the chemical diversity of volatile compounds (Booth et al. 2020). These findings indicate that both WGD and single-gene duplication events play important roles in the functional diversification of *TPS* genes, thereby contributing to the abundance and diversity of terpenoids in *L. tsaoko* and other plants. The significant accumulation of monoterpenoids in *L. tsaoko* fruits likely results from the diverse catalytic activities of monoTPS, where multiple TPSs are capable of producing the same monoterpenoids. For instance, LtTPS15 and LtTPS42 both synthesized *β*-myrcene. LtTPS20, LtTPS21, and LtTPS42 produced linalool. Compared to sesquiterpene synthases, there was a greater abundance of monoterpene synthases in *L. tsaoko*, with most capable of producing various types of monoterpenoids. This observation aligned with the higher detection rates of monoterpenoids in *L. tsaoko*. These results suggest that monoterpene synthases may play a dominant role in the biosynthesis of volatile compounds in *L. tsaoko.* Moreover, transcriptome analysis revealed predominant expression of *LtTPS* genes in flowers and fruits of *L. tsaoko*, correlating with the observed enrichment of volatile terpenoids in these tissues and corresponding enzyme activities. These findings underscore the pivotal role of these genes in shaping the profile and diversity of volatile terpenoids in *L. tsaoko* flowers and fruits.

The findings of this study provide a foundation for future research utilizing advanced technologies to further explore this topic. Recent technological developments in relevant fields present significant opportunities to elucidate the biosynthesis and regulation of active compounds in medicinal plants. Spatial metabolomics, for instance, can provide detailed mapping of bioactive compound localization within tissues, offering insights into their ecological and functional roles. Integrating metabolomics with transcriptomics, proteomics, and epigenomics facilitates a comprehensive, systems-level understanding of the pathways governing the synthesis and accumulation of key compounds. Emerging single-cell omics technologies further refine these approaches by identifying cell-specific metabolic activities (Zhang et al. 2023), while CRISPR-based functional genomics offers precise tools for gene function validation and pathway engineering. Collectively, these technologies possess substantial potential to illuminate the intricate biosynthetic networks in medicinal plants and to drive the development of innovative biotechnological strategies for enhancing valuable compound production.

## Materials and methods

### Sample collection and preparation

The root (R), stem (S), leaf (L), flower (Fl), fruit (Fr) and podetium (P) of *L. tsaoko* were collected from Honghe, Yunnan province. The collected materials were immediately weighed, frozen in liquid nitrogen, and stored at −80 °C until further processing. Subsequently, the samples were ground to a fine powder under cryogenic conditions using liquid nitrogen. Approximately 500 mg of the powder was then transferred to a 20 mL headspace vial (Agilent, Palo Alto, CA, USA) containing a saturated NaCl solution to inhibit enzymatic activity. The vials were sealed using crimp-top caps with TFE-silicone headspace septa (Agilent, Agilent Technologies, Santa Clara, CA, USA). For solid-phase microextraction (SPME) analysis, each sealed vial was incubated in a 60 °C water bath for 5 min. Following equilibration, a 120 µm DVB/CWR/PDMS fiber (Agilent, Agilent Technologies, Santa Clara, CA, USA) was inserted into the headspace of the sample for 15 min at 60 °C.

### GC–MS conditions

After sampling, desorption of the VOCs from the fibre coating was carried out in the injection port of the GC apparatus (Model 8890; Agilent, Agilent Technologies, Santa Clara, CA, USA) at 250 °C for 5 min in the splitless mode. The identification and quantification of VOCs were carried out using an Agilent Model 8890 GC and a 7000D mass spectrometer (Agilent, Agilent Technologies, Santa Clara, CA, USA), equipped with a 30 m × 0.25 mm × 0.25 μm DB-5MS (5% phenyl-polymethylsiloxane) capillary column. Helium was used as the carrier gas at a linear velocity of 1.2 mL⋅min^−1^. The injector temperature was kept at 250 °C and the detector at 280 °C. The oven temperature was programmed from 40 °C (3.5 min), increasing at 10 ℃⋅min^−1^ to 100 °C, at 7 ℃⋅min^−1^ to 180 °C, at 25 ℃⋅min^−1^ to 280 °C, and held for 5 min. Mass spectra were recorded in electron impact (EI) ionization mode at 70 eV. The quadrupole mass detector, ion source and transfer line temperatures were set, respectively, at 150, 230 and 280 °C. Selected ion monitoring (SIM) mode was used for the identification and quantification of analytes in GC–MS analysis. Each experiment was performed in three independent replicates.

### Qualitative, quantitative, quality control and statistical analysis of metabolites

The method described by Yuan et al. was used for the acquisition and processing of MS data (Yuan et al. 2022). Metabolite identification utilized the proprietary database of Wekemo company. Qualitative and quantitative analyses of the raw mass spectrometry data were conducted using MassHunter software (Zhu et al. 2013).

PCA, OPLS-DA, and hierarchical clustering analysis (HCA) were conducted using R software (https://www.r-project.org/) with the MetaboAnalyst package (Chong et al. 2018; Chong and Xia 2018). The correlation heatmap of metabolites was generated using R with the ggplot2 packages (Wickham 2011). VIP values (VIP ≥ 1) and fold change (FC) of metabolites (|Log2FC|≥ 1.0) were utilized to identify DAMs. The DAMs were subsequently mapped to the Kyoto Encyclopedia of Genes and Genomes (KEGG) pathway database (https://www.kegg.jp/kegg/pathway.html) for functional and pathway association analysis.

### Transcriptome library construction, sequencing and gene expression

Total RNA was extracted from six tissues (R, S, L, Fr, Fl, and P). The quality and purity of the RNA samples were evaluated using an RNA 6000 Nano LabChip Kit (Agilent Technologies, Santa Clara, CA, USA), with an RNA integrity number (RIN) of > 7.0. Poly-(A)-containing mRNA was isolated using a NEBNext® Poly(A) mRNA Magnetic Isolation Module (New England Biolabs, Ipswich, MA, USA). The mRNA was fragmented and used as templates to synthesize the first-strand cDNA using reverse transcriptase. Second-strand cDNA synthesis was performed using buffer and Second Strand Synthesis Enzyme Mix. The resulting double-stranded cDNA fragments underwent end-repair and adapter ligation. A USER enzyme excised uracil bases in the adapter. Adapter-modified fragments were selected through gel purification and amplified via PCR to generate the final cDNA library. The cDNA library preparation utilized the NEBNext® Ultra™ RNA Library Prep Kit for Illumina® (New England Biolabs, Ipswich, MA, USA). Following this methodology, six libraries representing different tissues were constructed, with each experiment conducted in triplicate. These libraries were sequenced on the NovaSeq 6000 platform (Illumina, San Diego, CA, USA). Raw reads underwent quality control using Trimmomatic (Bolger et al. 2014), and clean reads were aligned to the reference genome (https://doi.org/10.6084/m9.figshare.27600624.v1) using HISAT2 (Kim et al. 2015). The *L. tsaoko* genome assembly and annotation were previously completed by our research group. Gene expression levels were quantified as fragments per kilobase of transcript per million fragments mapped (FPKM) using StringTie 2 (Kovaka et al. 2019). Data normalization was performed using TBtools (Chen et al. 2018), employing the *z*-score method.

### Identification of genes related to terpenoid biosynthesis

To investigate the genes involved in terpenoid skeleton synthesis pathways, we initially retrieved protein sequences from the *Arabidopsis thaliana* genome, including 1-deoxy-D-xylulo se-5-phosphate synthase (DXS), 1-deoxy-D-xylulose-5-phosphate reductoisomerase (DXR), 2-Cmethyl-D-erythritol 4-phosphate cytidylyltransferase (MCT), 4-diphosphocytidyl-2-C-methyl-D-erythritol kinase (CMK), 2-C-methyl-D-erythritol-2,4-cyclodiphosphate synthase (MCS), (E)−4-hydroxy-3-methylbut-2-enyl-diphosphate synthase (HDS), 4-hydroxy-3-methylbut-2-enyl-diphosphate reductase (HDR), acyl-coenzyme A-cholesterol acyltransferase (ACAT), hydroxymethylglutaryl-CoA synthase (HMGS), hydroxymethylglutaryl-CoA reductase (HMGR), mevalonate kinase (MVK), phosphomevalonate kinase (PMK), mevalonate diphosphate decarboxylase (MVD), isopentenyl-diphosphate isomerase (IDI), geranyl diphosphate synthase (GPPS), and farnesyl diphosphate synthase (FPPS) from the NCBI database. We created a BLAST database for the selected sequences using the makeblastdb tools of BLAST + . These sequences served as queries and were blasted against the protein sequences of *L. tsaoko* with an *E*-value cutoff of 1e^−5^. Candidates with over 40% identity and alignment protein length exceeding 200 amino acids were selected for further analysis.

To identify candidate TPS genes, we employed Hidden Markov Model (HMM) profiles of Terpene_synth (PF01397) and Terpene_synth_C (PF03936) obtained from the Pfam database (http://pfam.xfam.org). These profiles were subsequently used to search against *L. tsaoko* protein sequences using HMMER (Johnson et al., 2010), with an *E*-value threshold of 1e^−5^. To categorize the TPS genes into distinct subfamilies, we acquired TPS protein sequences from various plants, including *Vitis vinifera*, *Oryza sativa*, *A. thaliana*, *Populus trichocarpa*, *Sorghum bicolor*, *Selaginella tamariscina* and *Physcomitrium patens* (Chen et al. 2011). Subsequently, the TPS sequences from different species and *L.tsaoko* were aligned using MAFFT, and a maximum likelihood tree was constructed using IQ-TREE (Nguyen et al. 2015) with 1000 bootstrap replicates. The visual enhancement of the phylogenetic tree was performed using ITOL (https://itol.embl.de/). Different modes of gene duplication in *L. tsaoko* were classified into five categories using DupGen_Finder (Qiao et al. 2019) (v1.12) with default parameters.

### Gene structure analysis, motif identification and chromosomal locations of *LtTPS*

The length and chromosomal location of *LtTPSs* were derived from the *L. tsaoko* genome annotation file. Chromosomal mapping of genes was conducted using TBtools (Chong and Xia 2018). The Gene Structure Display Server 2.0 (http://gsds.gao-lab.org/) was utilized to determine the intron and exon structures of *LtTPS*s using genomic and cDNA sequences (Hu et al. 2015). Protein motif analysis of LtTPSs was performed using the online MEME Suite (http://meme-suite.org/) with default parameters and a set motif number of twenty. CDD (https://www.ncbi.nlm.nih.gov/Structure/bwrpsb/bwrpsb.cgi) was employed to identify conserved domains within the predicted TPS protein sequences.

### Gene clone and functional identification of *LtTPSs*

Several *LtTPS* genes identified in this study were manually refined due to the presence of long introns and multiple exons using Softberry (https://www.softberry.com). mRNA was extracted from *L. tsaoko* composite samples across six tissues RNAprep Pure Plant Plus Kit (TIANGEN Biotech (Beijing, China)), and cDNA was isolated using the Reverse Transcription System (Promega, Madison, WI, USA) according to the manufacturer's protocol. Specific primers (Table S8) were designed by TaKaRa (https://www.takarabio.com/learning-centers/cloning/primer-design-and-other-tools). *LtTPSs* were subsequently cloned into pET28a/ pRSFDuet-MispA vectors (Lei et al. 2021). Recombinant pET28a-LtsaTPSs plasmids and pMevT-MBIS (Glasscock et al. 2021) were transformed into *Escherichia coli* strain BL21 (DE3) for sesquiterpene protein expression. pRSFDuet-MispA-LtTPSs were transformed into *E. coli* strain EIP81 (DE3) (Lei et al. 2021) for monoterpene protein expression. The transformants were inoculated into 5 mL of TB medium and cultivated overnight at 37 °C and 200 rpm. A 2 mL aliquot of each preculture was transferred to 50 mL of fresh TB medium in a flask and incubated with shaking (200 rpm) at 37 °C. Protein expression was induced by 1 mM of Isopropyl *β*-D-thiogalactopyranoside (IPTG) and 1 mM MgCl_2_ when the optical density at 600 nm (OD_600_) reached 0.6 − 0.8, followed by incubation at 16 °C for an additional 76 h. A 2 mL sample of induced bacterial culture was collected for GC analysis.

GC analysis was conducted using an Agilent System 8890 GC/Q-tof equipped with a flame ionization detector (FID) and an HP-5MS UI column (15 m × 0.25 mm, 0.25 μm film thickness). The headspace extraction vial was equilibrated at 50 °C for 10 min (rotational speed 300 rpm). The Carbon WR/PDMS SPME Arrow underwent conditioning at 250 °C for 10 min, adsorption for 40 min, and desorption for 10 min. The inlet temperature was set to 250 °C. The column temperature program was initiated at 40 °C for 5 min, then increased at 2 ℃⋅min^−1^ to 120 °C, and finally raised at 10 ℃⋅min^−1^ to 290 °C, where it was maintained for 29 min. Mass spectrometry scanning was performed within the mass range of 35 m*/z* to 500 m*/z*, with a scan rate of 781 u/s.

## Supplementary Information

Supplementary Material 1.

Supplementary Material 2.

## Data Availability

The datasets generated and analyzed during the current study are available in the Sequence Read Archive (SRA), Biological Research Project Data (BioProject), NCBI repository, accession: PRJNA1171101. The reference genome files and annotation files are accessible through Figshare (https://doi.org/10.6084/m9.figshare.27600624.v1).

## Abbreviations

MVA: Mevalonate
MEP: Plastidial 2-C-methyl-D-erythritol-4-phosphate
FPP: Farnesyl diphosphate
GPP: Geranyl diphosphate
DXS: 1-Deoxy-D-xylulose-5-phosphate synthase
GPPS: Geranyl diphosphate synthase
IDI: Isopentenyl diphosphate isomerase
FPPS: Farnesyl diphosphate synthase
DXR: 1-Deoxy-D-xylulose-5-phosphate reductase
PKS: Polyketide synthase
DMAPP: Dimethylallyl diphosphate
IPP: Isopentenyl diphosphate
MCT: 2-C-Methyl-D-erythritol 4-phosphate Cytidylyltransferase
CMK: 4-Diphosphocytidyl-2-C-methyl-D-erythritol kinase
MDS: 2-C-methyl-D-erythritol2,4-cyclodiphosphate synthase
HMGS: Hydroxymethylglutaryl-CoA synthase
HMGR: Hydroxymethylglutaryl-CoA reductase
HDS: (E)-4-hydroxy-3-methylbut-2-enyl-diphosphate synthase
MVK: Mevalonate kinase
MVD: Mevalonate diphosphate decarboxylase
PMK: Phosphomevalonate kinase
TPS: Terpene synthase
GC/MS: Gas chromatography-mass spectrometry
PCA: Principal component analysis
VOCs: Volatile organic compounds
DAMs: Differentially Accumulated Metabolites
VIP: Variable Importance in Projection
KEGG: Kyoto Encyclopedia of Genes and Genomes
GRAVY: Grand average of hydropathicity
pI: Isoelectric point
MW: Molecular weight
SPME: Solid-phase microextraction
HCA: Hierarchical clustering analysis
OPLS-DA: Orthogonal Partial Least Squares Discriminant Analysis

## References

[CR1] Ahmad N, Xu Y, Zang F, Li D, Liu Z. The evolutionary trajectories of specialized metabolites towards antiviral defense system in plants. Mol Hortic. 2024;4:2. 38212862 10.1186/s43897-023-00078-9 PMC10785382

[CR2] Alicandri E, Paolacci AR, Osadolor S, Sorgonà A, Badiani M, Ciaffi M. On the evolution and functional diversity of terpene synthases in the *Pinus* species: a review. J Mol Evol. 2020;88:253–83. 32036402 10.1007/s00239-020-09930-8

[CR3] Allen KD, McKernan K, Pauli C, Roe J, Torres A, Gaudino R. Genomic characterization of the complete terpene synthase gene family from *Cannabis sativa*. PLoS ONE. 2019;14: e0222363. 31513654 10.1371/journal.pone.0222363 PMC6742361

[CR4] Alquézar B, Rodríguez A, de la Peña M, Peña L. Genomic analysis of terpene synthase family and functional characterization of seven sesquiterpene synthases from *Citrus sinensis*. Front Plant Sci. 2017;8:1481. 28883829 10.3389/fpls.2017.01481 PMC5573811

[CR5] Bolger AM, Lohse M, Usadel B. Trimmomatic: a flexible trimmer for Illumina sequence data. Bioinformatics. 2014;30:2114–20. 24695404 10.1093/bioinformatics/btu170 PMC4103590

[CR6] Booth JK, Yuen MM, Jancsik S, Madilao LL, Page JE, Bohlmann J. Terpene synthases and terpene variation in *Cannabis sativa*. Plant Physiol. 2020;184:130–47. 32591428 10.1104/pp.20.00593 PMC7479917

[CR7] Cao P, Yang J, Xia L, et al. Two gene clusters and their positive regulator SlMYB13 that have undergone domestication-associated negative selection control phenolamide accumulation and drought tolerance in tomato. Mol Plant. 2024;17:579–97. 38327054 10.1016/j.molp.2024.02.003

[CR8] Chaw S-M, Liu Y-C, Wu Y-W, et al. *Stout camphor* tree genome fills gaps in understanding of flowering plant genome evolution. Nat Plants. 2019;5:63–73. 30626928 10.1038/s41477-018-0337-0 PMC6784883

[CR9] Chen F, Tholl D, Bohlmann J, Pichersky E. The family of terpene synthases in plants: a mid-size family of genes for specialized metabolism that is highly diversified throughout the kingdom. Plant J. 2011;66:212–29. 21443633 10.1111/j.1365-313X.2011.04520.x

[CR10] Chen C, Chen H, He Y, Xia R. TBtools, a toolkit for biologists integrating various biological data handling tools with a user-friendly interface. BioRxiv. 2018;289660: 289660.

[CR11] Chen Y-C, Li Z, Zhao Y-X, et al. The *Litsea* genome and the evolution of the laurel family. Nat Commun. 2020;11:1675. 32245969 10.1038/s41467-020-15493-5 PMC7125107

[CR12] Chong J, Xia J. MetaboAnalystR: an R package for flexible and reproducible analysis of metabolomics data. Bioinformatics. 2018;34:4313–4. 29955821 10.1093/bioinformatics/bty528 PMC6289126

[CR13] Chong J, Soufan O, Li C, Caraus I, Li S, Bourque G, Wishart DS, Xia J. MetaboAnalyst 4.0: towards more transparent and integrative metabolomics analysis. Nucleic Acids Res. 2018;46:W486–94. 29762782 10.1093/nar/gky310 PMC6030889

[CR14] Christianson DW. Structural and chemical biology of terpenoid cyclases. Chem Rev. 2017;117:11570–648. 28841019 10.1021/acs.chemrev.7b00287 PMC5599884

[CR15] Dai M, Peng C, Peng F, Xie C, Wang P, Sun F. Anti-Trichomonas vaginalis properties of the oil of *Amomum tsao-ko* and its major component, geraniol. Pharm Biol. 2016;54:445–50. 25963227 10.3109/13880209.2015.1044617

[CR16] Dudareva N, Negre F, Nagegowda DA, Orlova I. Plant volatiles: recent advances and future perspectives. Crit Rev Plant Sci. 2006;25:417–40.

[CR17] Feng X, Jiang Z-T, Wang Y, Li R. Composition comparison of essential oils extracted by hydrodistillation and microwave-assisted hydrodistillation from *Amomum tsaoko* in China. J Essent Oil Bearing Plants. 2010;13:286–91.

[CR18] Gao Y, Honzatko RB, Peters RJ. Terpenoid synthase structures: a so far incomplete view of complex catalysis. Nat Prod Rep. 2012;29:1153–75. 22907771 10.1039/c2np20059g PMC3448952

[CR19] Gershenzon J, Dudareva N. The function of terpene natural products in the natural world. Nat Chem Biol. 2007;3:408–14. 17576428 10.1038/nchembio.2007.5

[CR20] Glasscock CJ, Biggs BW, Lazar JT, et al. Dynamic control of gene expression with riboregulated switchable feedback promoters. ACS Synth Biol. 2021;10:1199–213. 33834762 10.1021/acssynbio.1c00015 PMC8141045

[CR21] He X-F, Zhang X-K, Geng C-A, Hu J, Zhang X-M, Guo Y-Q, Chen J-J. Tsaokopyranols A-M, 2, 6-epoxydiarylheptanoids from *Amomum tsao-ko* and their α-glucosidase inhibitory activity. Bioorg Chem. 2020;96:103638. 10.1016/j.bioorg.2020.103638 32062448

[CR22] Holopainen JK, Gershenzon J. Multiple stress factors and the emission of plant VOCs. Trends Plant Sci. 2010;15:176–84. 20144557 10.1016/j.tplants.2010.01.006

[CR23] Hu B, Jin J, Guo A-Y, Zhang H, Luo J, Gao G. GSDS 2.0: an upgraded gene feature visualization server. Bioinformatics. 2015;31:1296–7. 25504850 10.1093/bioinformatics/btu817 PMC4393523

[CR24] Johnson LS, Eddy SR, Portugaly E. Hidden Markov model speed heuristic and iterative HMM search procedure. BMC Bioinform. 2010;11:431. 10.1186/1471-2105-11-431 PMC2931519 20718988

[CR25] Jun Yang RC, Wang C, Li C, Ye W, Zhang Z, Wang S. A widely targeted metabolite modificomics strategy for modified metabolites identification in tomato. J Integr Plant Biol. 2024;66:810–23. 38375781 10.1111/jipb.13629

[CR26] Karunanithi PS, Zerbe P. Terpene synthases as metabolic gatekeepers in the evolution of plant terpenoid chemical diversity. Front Plant Sci. 2019;10:1166. 31632418 10.3389/fpls.2019.01166 PMC6779861

[CR27] Kim D, Langmead B, Salzberg SL. HISAT: a fast spliced aligner with low memory requirements. Nat Methods. 2015;12:357–60. 25751142 10.1038/nmeth.3317 PMC4655817

[CR28] Kohzaki K, Gomi K, Yamasaki-Kokudo Y, Ozawa R, Takabayashi J, Akimitsu K. Characterization of a sabinene synthase gene from rough lemon (*Citrus jambhiri*). J Plant Physiol. 2009;166:1700–4. 19433341 10.1016/j.jplph.2009.04.003

[CR29] Kovaka S, Zimin AV, Pertea GM, Razaghi R, Salzberg SL, Pertea M. Transcriptome assembly from long-read RNA-seq alignments with StringTie2. Genome Biol. 2019;20:278. 31842956 10.1186/s13059-019-1910-1 PMC6912988

[CR30] Kubeczka, K.-H. History and sources of essential oil research. In Handbook of essential oils (CRC Press). 2020. pp. 3–39.

[CR31] Lei D, Qiu Z, Wu J, Qiao B, Qiao J, Zhao GR. Combining Metabolic and Monoterpene Synthase Engineering for de Novo Production of Monoterpene Alcohols in *Escherichia coli*. ACS Synth Biol. 2021;10:1531–44. 34100588 10.1021/acssynbio.1c00081

[CR32] Liang M, Wu Y, Wang R, Zhang Z, Xin R, Liu Y. Insights into the key odorants in fresh and dried *Amomum tsaoko* using the sensomics approach. Food Chemistry: X. 2024;22:101344. 38595757 10.1016/j.fochx.2024.101344 PMC11002797

[CR33] Lichtenthaler HK. The 1-deoxy-D-xylulose-5-phosphate pathway of isoprenoid biosynthesis in plants. Annu Rev Plant Biol. 1999;50:47–65. 10.1146/annurev.arplant.50.1.47 15012203

[CR34] Liu H, Yan Q, Zou D, et al. Identification and bioactivity evaluation of ingredients from the fruits of *Amomum tsaoko* Crevost et Lemaire. Phytochem Lett. 2018;28:111–5.

[CR35] Liu S, Yang S, Su P. Chemo-enzymatic synthesis of bioactive compounds from traditional Chinese medicine and medicinal plants. Science of Traditional Chinese Medicine. 2024;10:1097.

[CR36] Lücker J, El Tamer MK, Schwab W, Verstappen FW, van der Plas LH, Bouwmeester HJ, Verhoeven HA. Monoterpene biosynthesis in lemon (*Citrus limon*) cDNA isolation and functional analysis of four monoterpene synthases. Eur J Biochem. 2002;269:3160–71. 12084056 10.1046/j.1432-1033.2002.02985.x

[CR37] Myburg AA, Grattapaglia D, Tuskan GA, et al. The genome of *Eucalyptus grandis*. Nature. 2014;510:356–62. 24919147 10.1038/nature13308

[CR38] Nagegowda DA, Gupta P. Advances in biosynthesis, regulation, and metabolic engineering of plant specialized terpenoids. Plant Sci. 2020;294: 110457. 32234216 10.1016/j.plantsci.2020.110457

[CR39] Nes WD. Biosynthesis of cholesterol and other sterols. Chem Rev. 2011;111:6423–51. 21902244 10.1021/cr200021m PMC3191736

[CR40] Newman JD, Chappell J. Isoprenoid biosynthesis in plants: carbon partitioning within the cytoplasmic pathway. Crit Rev Biochem Mol Biol. 1999;34:95–106. 10333387 10.1080/10409239991209228

[CR41] Nguyen L-T, Schmidt HA, Von Haeseler A, Minh BQ. IQ-TREE: a fast and effective stochastic algorithm for estimating maximum-likelihood phylogenies. Mol Biol Evol. 2015;32:268–74. 25371430 10.1093/molbev/msu300 PMC4271533

[CR42] Pichersky E, Raguso RA. Why do plants produce so many terpenoid compounds? New Phytol. 2018;220:692–702. 27604856 10.1111/nph.14178

[CR43] Qiao X, Li Q, Yin H, Qi K, Li L, Wang R, Zhang S, Paterson AH. Gene duplication and evolution in recurring polyploidization–diploidization cycles in plants. Genome Biol. 2019;20:38. 30791939 10.1186/s13059-019-1650-2 PMC6383267

[CR44] Qiao Z, Hu H, Shi S, Yuan X, Yan B, Chen L. An update on the function, biosynthesis and regulation of floral volatile terpenoids. Horticulturae. 2021;7:451.

[CR45] Rawat A, Prakash O, Nagarkoti K, Kumar R, Negi MS, Kumar S, Srivastava RM. Chemical profiling and bioactivity evaluation of thymol rich *Coleus aromaticus* Benth. essential oil. Med Plant Biol. 2024;3:e007

[CR46] Schnee C, Köllner TG, Held M, Turlings TC, Gershenzon J, Degenhardt J. The products of a single maize sesquiterpene synthase form a volatile defense signal that attracts natural enemies of maize herbivores. Proc Natl Acad Sci. 2006;103:1129–34. 16418295 10.1073/pnas.0508027103 PMC1347987

[CR47] Shi S, Luo Y, Ma Y, Chu Y, Wang Y, Chen X, Chu Y. Identification of in vitro-in vivo components of Caoguo using accelerated solvent extraction combined with gas chromatography-mass spectrometry integrated with network pharmacology on indigestion. Ann Transl Med. 2021;9(15):1247. 10.21037/atm-21-3245 PMC8421984 34532384

[CR48] Shimada T, Endo T, Fujii H, Hara M, Ueda T, Kita M, Omura M. Molecular cloning and functional characterization of four monoterpene synthase genes from *Citrus unshiu* Marc. Plant Sci. 2004;166:49–58.

[CR49] Tholl D. Biosynthesis and biological functions of terpenoids in plants. Adv Biochem Eng Biotechnol. 2015;148:63–106. 10.1007/10_2014_295 25583224

[CR50] Wang Q, Quan S, Xiao H. Towards efficient terpenoid biosynthesis: manipulating IPP and DMAPP supply. Bioresources and Bioprocessing. 2019a;6:6.

[CR51] Wang S, Ouyang K, Wang K. Genome-wide identification, evolution, and expression analysis of TPS and TPP gene families in *Brachypodium distachyon*. Plants. 2019b;8:362. 31547557 10.3390/plants8100362 PMC6843561

[CR52] Wang X, Gao Y, Wu X, et al. High-quality evergreen azalea genome reveals tandem duplication-facilitated low-altitude adaptability and floral scent evolution. Plant Biotechnol J. 2021;19:2544–60. 34375461 10.1111/pbi.13680 PMC8633516

[CR53] Wang W, Wang MY, Zeng Y, Chen X, Wang X, Barrington AM, Tao J, Atkinson RG, Nieuwenhuizen NJ. The terpene synthase (TPS) gene family in kiwifruit shows high functional redundancy and a subset of TPS likely fulfil overlapping functions in fruit flavour, floral bouquet and defence. Mol Hortic. 2023;3:9. 37789478 10.1186/s43897-023-00057-0 PMC10514967

[CR54] Wang Q, Jiang J, Liang Y, Li S, Xia Y, Zhang L, Wang X. Expansion and functional divergence of terpene synthase genes in angiosperms: a driving force of terpene diversity. Hort Res. 2024;uhae272. 10.1093/hr/uhae272 PMC11725647 39897732

[CR55] Wei Z, Bingyue L, Hengling M, Xiang W, Zhiqing Y, Shengchao Y. Phenotypic diversity analysis of the fruit of *Amomum tsao-ko* Crevost et Lemarie, an important medicinal plant in Yunnan, China. Genet Resour Crop Evol. 2019;66:1145–54.

[CR56] Wei Y, Zhang J, Qi K, Li Y, Chen Y. Combined analysis of transcriptomics and metabolomics revealed complex metabolic genes for diterpenoids biosynthesis in different organs of *Anoectochilus roxburghii*. Chinese Herb Med. 2023;15:298–309. 10.1016/j.chmed.2022.11.002 PMC10230631 37265764

[CR57] Wickham H. ggplot2. Wiley Interdisciplinary Reviews: Computational Statistics. 2011;3:180–5.

[CR58] Wu X, Yang Y, Wang M, Shao C, Morillas JIV, Yuan F, liu J, Zhang H. Improving coriander yield and quality with a beneficial bacterium. Mol Hortic. 2024;4:8. 10.1186/s43897-024-00087-2 PMC10903023 38419111

[CR59] Yadav P, Mohapatra S, Jaiswal PO, Dokka N, Tyagi S, Sreevathsa R, Shasany AK. Characterization of a novel cytosolic sesquiterpene synthase MpTPS4 from *Menth*a ×*piperita* as a bioresource for the enrichment of invaluable viridiflorol in mentha essential oil. Int J Biol Macromol. 2024;277:134214. 10.1016/j.ijbiomac.2024.134214 39069055

[CR60] Yang Y, Yan RW, Cai XQ, Zheng ZL, Zou GL. Chemical composition and antimicrobial activity of the essential oil of *Amomum tsao-ko*. J Sci Food Agric. 2008;88:2111–6.

[CR61] Yang P, Zhao HY, Wei JS, et al. Chromosome-level genome assembly and functional characterization of terpene synthases provide insights into the volatile terpenoid biosynthesis of *Wurfbainia villosa*. Plant J. 2022;112:630–45. 36071028 10.1111/tpj.15968

[CR62] Yang SM, Chu HY, Wang YX, Guo BL, An TY, Shen Q. Analysis of monoterpene biosynthesis and functional TPSs of *Perilla frutescens *based on transcriptome and metabolome. Med Plant Biol. 2024;3.e017

[CR63] Yuan H, Cao G, Hou X, et al. Development of a widely targeted volatilomics method for profiling volatilomes in plants. Mol Plant. 2022;15:189–202. 34509640 10.1016/j.molp.2021.09.003

[CR64] Zerbe P, Bohlmann J. Plant diterpene synthases: exploring modularity and metabolic diversity for bioengineering. Trends Biotechnol. 2015;33:419–28. 26003209 10.1016/j.tibtech.2015.04.006

[CR65] Zhang J, Ahmad M, Gao H. Application of single-cell multi-omics approaches in horticulture research. Mol Hortic. 2023;3:18. 37789394 10.1186/s43897-023-00067-y PMC10521458

[CR66] Zhao Y, Liu Y, Chen Y, Gao M, Wu L, Wang Y. Overexpression of 1-deoxy-D-xylulose-5-phosphate reductoisomerase enhances the monoterpene content in *Litsea cubeba*. For Res. 2023a;3:11. 10.48130/FR-2023-0011 PMC11524321 39526280

[CR67] Zhao Y, Liu G, Yang F, et al. Multilayered regulation of secondary metabolism in medicinal plants. Mol Hortic. 2023b;3(1):11. 37789448 10.1186/s43897-023-00059-y PMC10514987

[CR68] Zhou F, Pichersky E. The complete functional characterisation of the terpene synthase family in tomato. New Phytol. 2020;226:1341–60. 31943222 10.1111/nph.16431 PMC7422722

[CR69] Zhou W, Huang F, Li S, et al. Molecular cloning and characterization of two 1-deoxy-D-xylulose-5-phosphate synthase genes involved in tanshinone biosynthesis in *Salvia miltiorrhiza*. Mol Breed. 2016;36:124.

[CR70] Zhu ZJ, Schultz AW, Wang J, Johnson CH, Yannone SM, Patti GJ, Siuzdak G. Liquid chromatography quadrupole time-of-flight mass spectrometry characterization of metabolites guided by the METLIN database. Nat Protoc. 2013;8:451–60. 23391889 10.1038/nprot.2013.004 PMC3666335

