# Supplementary material for: Comprehensive characterization of volatile terpenoids and terpene synthases in Lanxangia tsaoko
Source: Mol Hortic. 2025 Apr 3;5:20. doi: 10.1186/s43897-024-00140-0 (PMC11966916; doi:10.1186/s43897-024-00140-0)
Supplement: Supplementary file 1 — Supplementary Material 1. [file 43897_2024_140_MOESM1_ESM.docx]

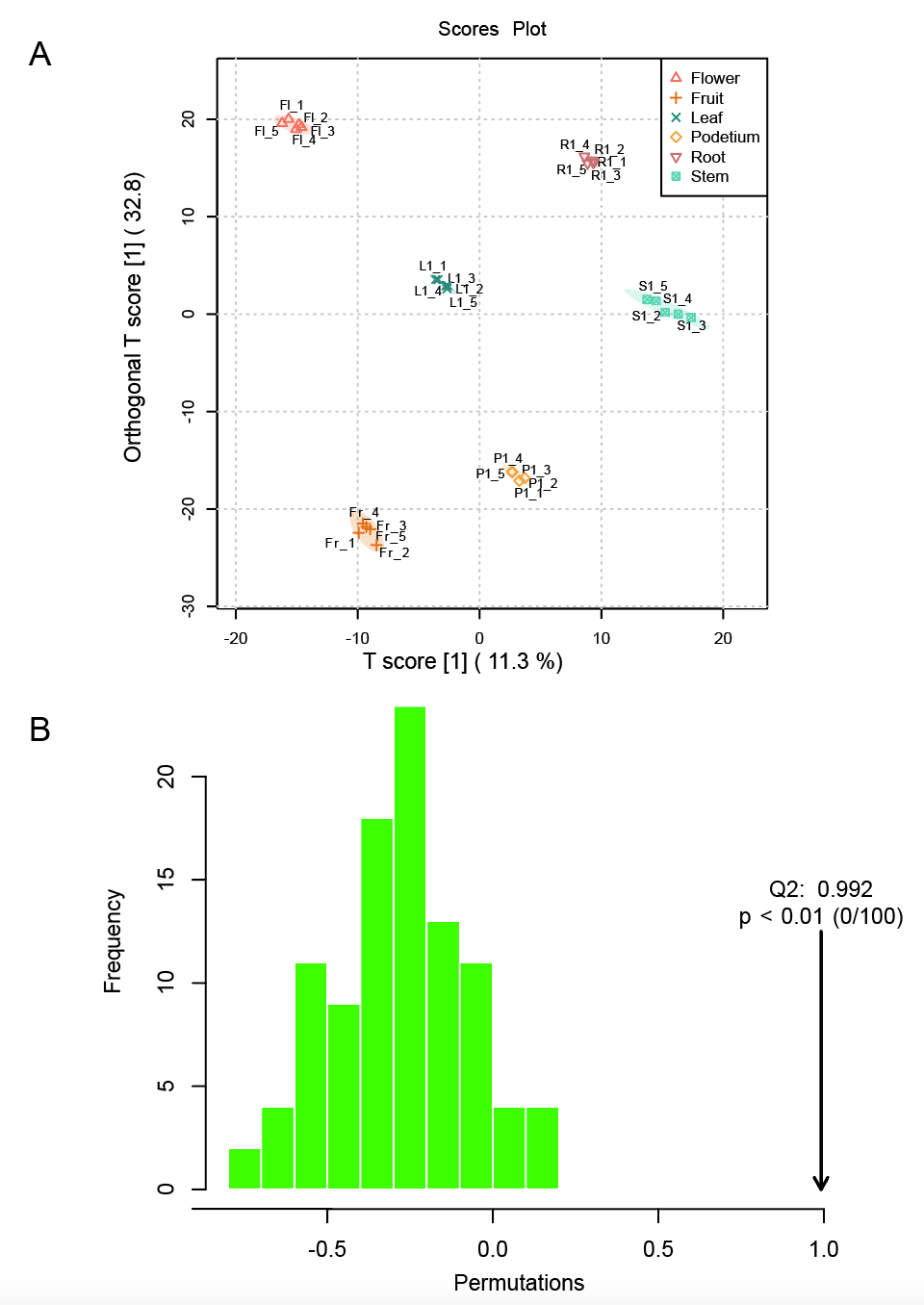


Figure S1. OPLS-DA score plot and cross-validation plot. **A.** Score plots of the OPLS-DA model. Each point represents a sample, points of the same shape represent the same tissue and the separation between the groups indicates class distinction. B. Distribution of the test statistic (Q2) for the OPLS-DA permutation test and the p-value.


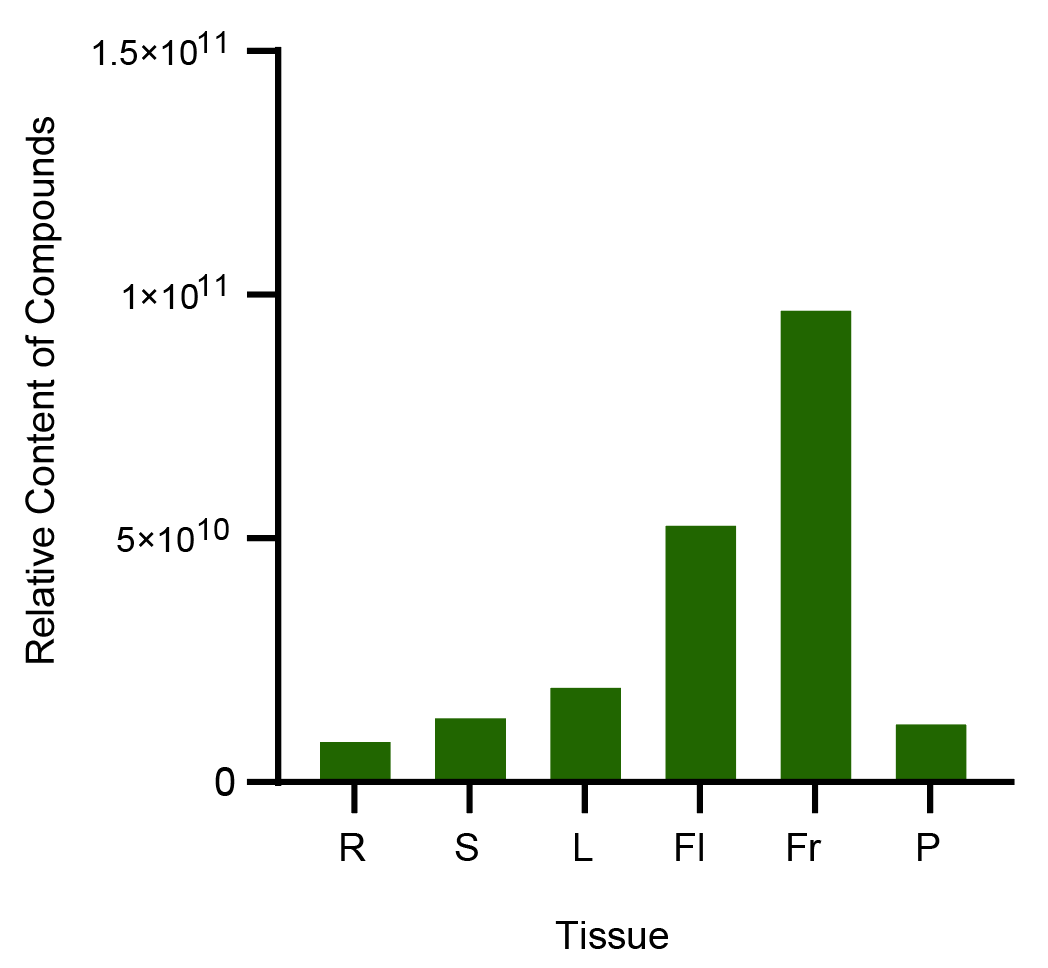


Figure S2. Relative content of all volatile compounds in different tissues. R: root, S: stem, L: leaf, Fl: flower, Fr: fruit, P: podetium.


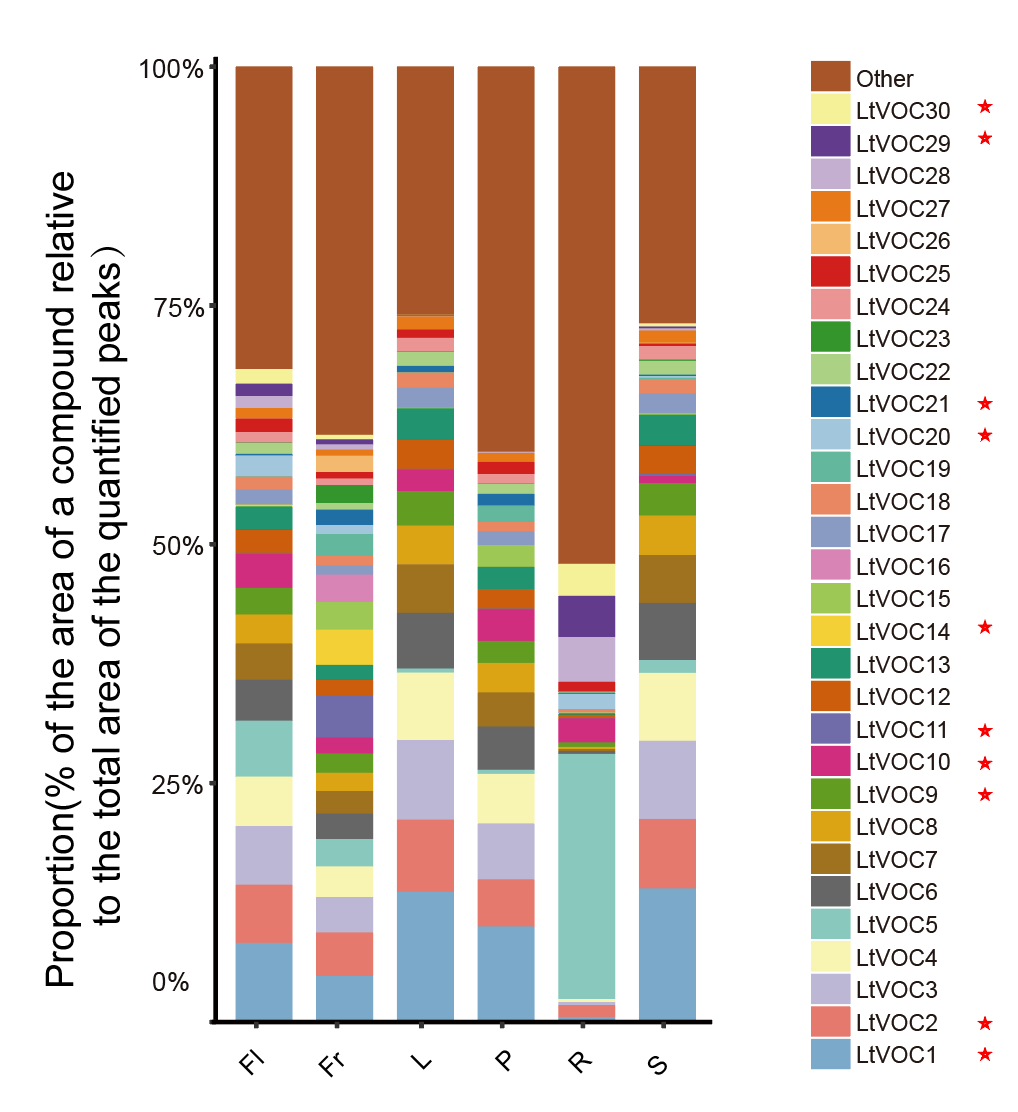


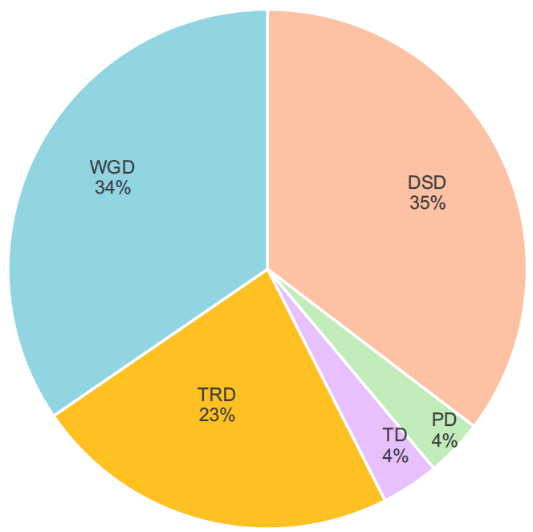
Figure S3. Percentage stacked bar chart of top 30 volatile compounds in six tissues. Compounds marked with an asterisk belong to the terpenoid.

Figure S4. Pie diagram shows the proportation of different modes of gene duplication. WGD: whole-genome duplication, TD: tandem duplication, PD: proximal duplication, TRD: transposed duplication, DSD: dispersed duplication.
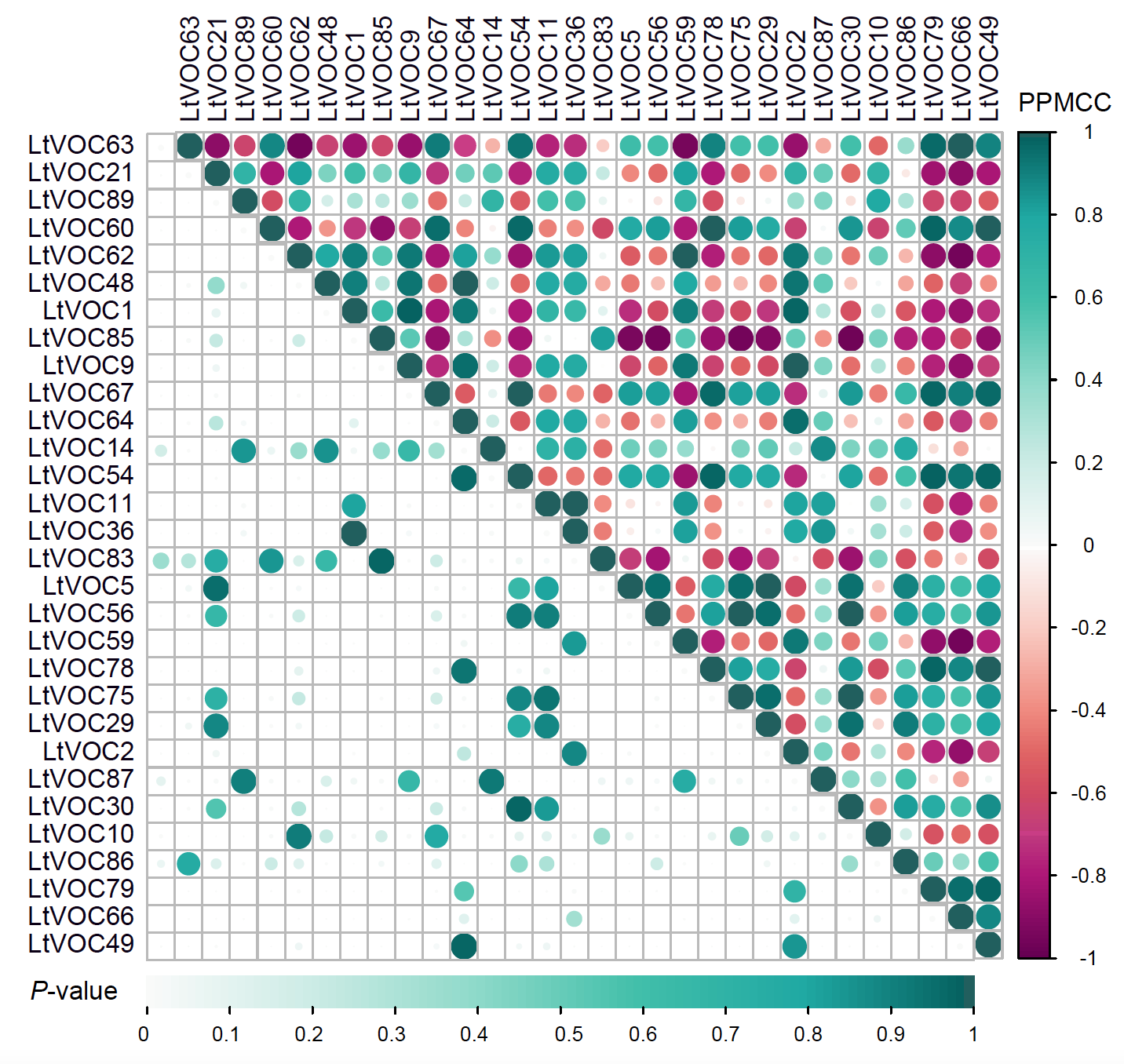


Figure S5. Visual representation of the pearson correlation matrix of the top 30 volatile terpenoids. The triangular matrix in the upper right corner is the Pearson correlation and the triangular matrix in the lower left corner is the p-value. The vertical color bar represents the strength of the correlation between the metabolites and horizontal color bar represents the p-value. Darker blue color and larger dot size indicate stronger positive correlation and higher p-value, while darker red means stronger negative correlation (PPMCC: Pearson Product-Moment Correlation Coefficient)


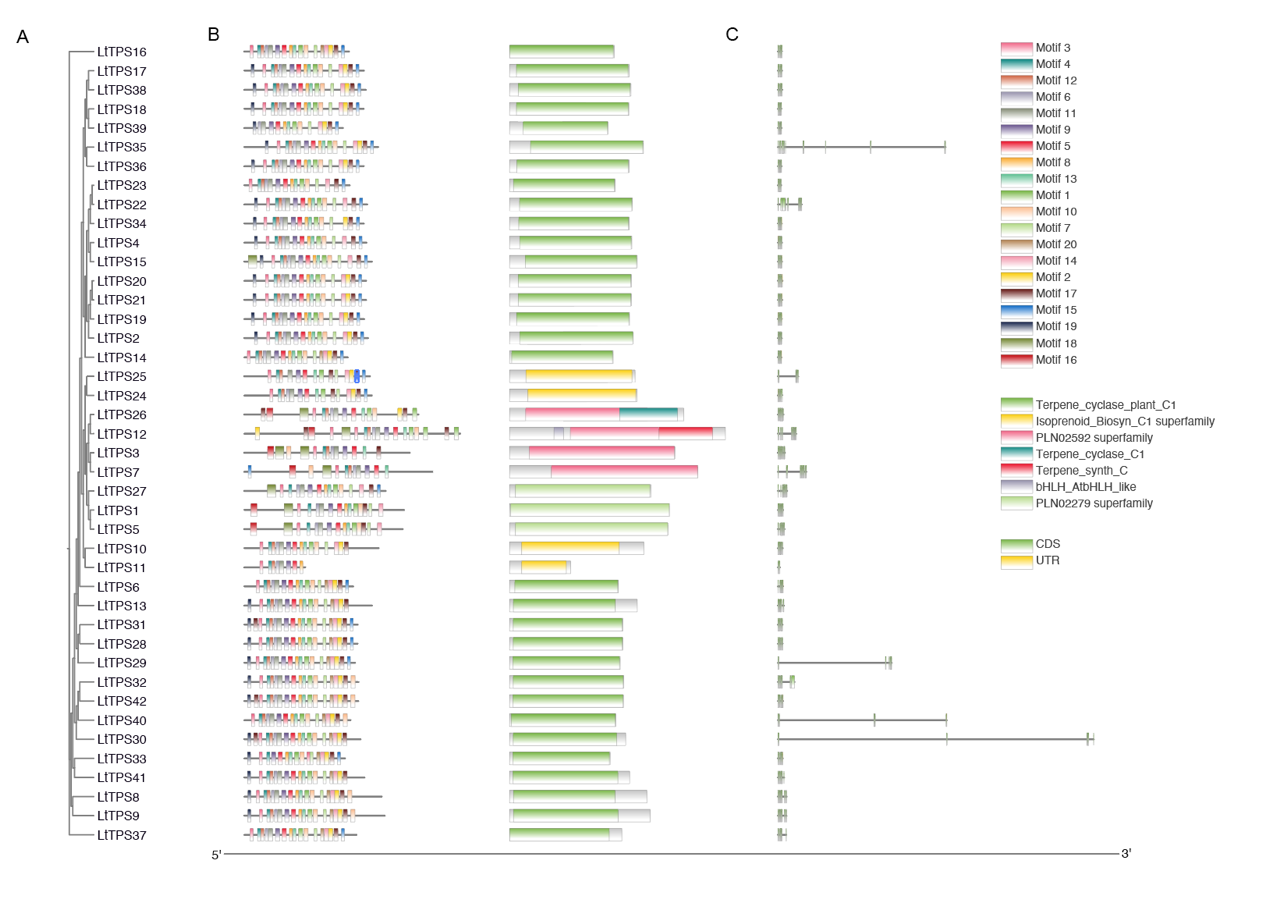
Figure S6. Phylogenetic relationships, gene structure, and architecture of conserved protein motifs of the *LtTPS* genes. **A.** The phylogenetic tree was constructed based on the full-length sequences of LtTPS proteins using IQ-TREE. **B.** Motif compositions of the LtTPS proteins are illustrated, with motifs numbered 1–20 displayed in distinct colored boxes. The sequence information for each motif is provided in Table S3. Protein lengths can be estimated using the scale at the bottom. **C.** Exon-intron structures of *LtTPS* genes are presented, with green boxes representing untranslated 5′- and 3′-regions and black lines indicating introns.


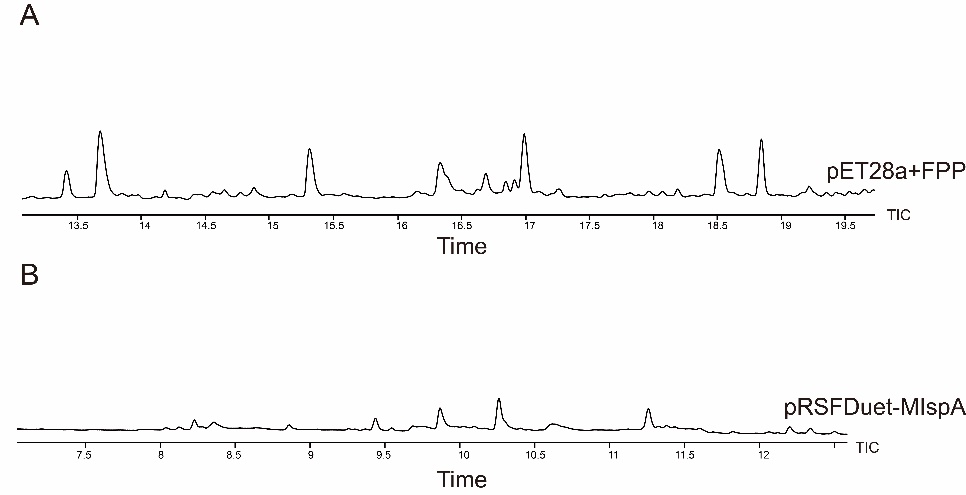


Figure S7. Characterization of control. **A.** GC-MS total ion chromatograms of pET28a with FPP. **B.** GC-MS total ion chromatograms of pRSFDuet-MIspA.


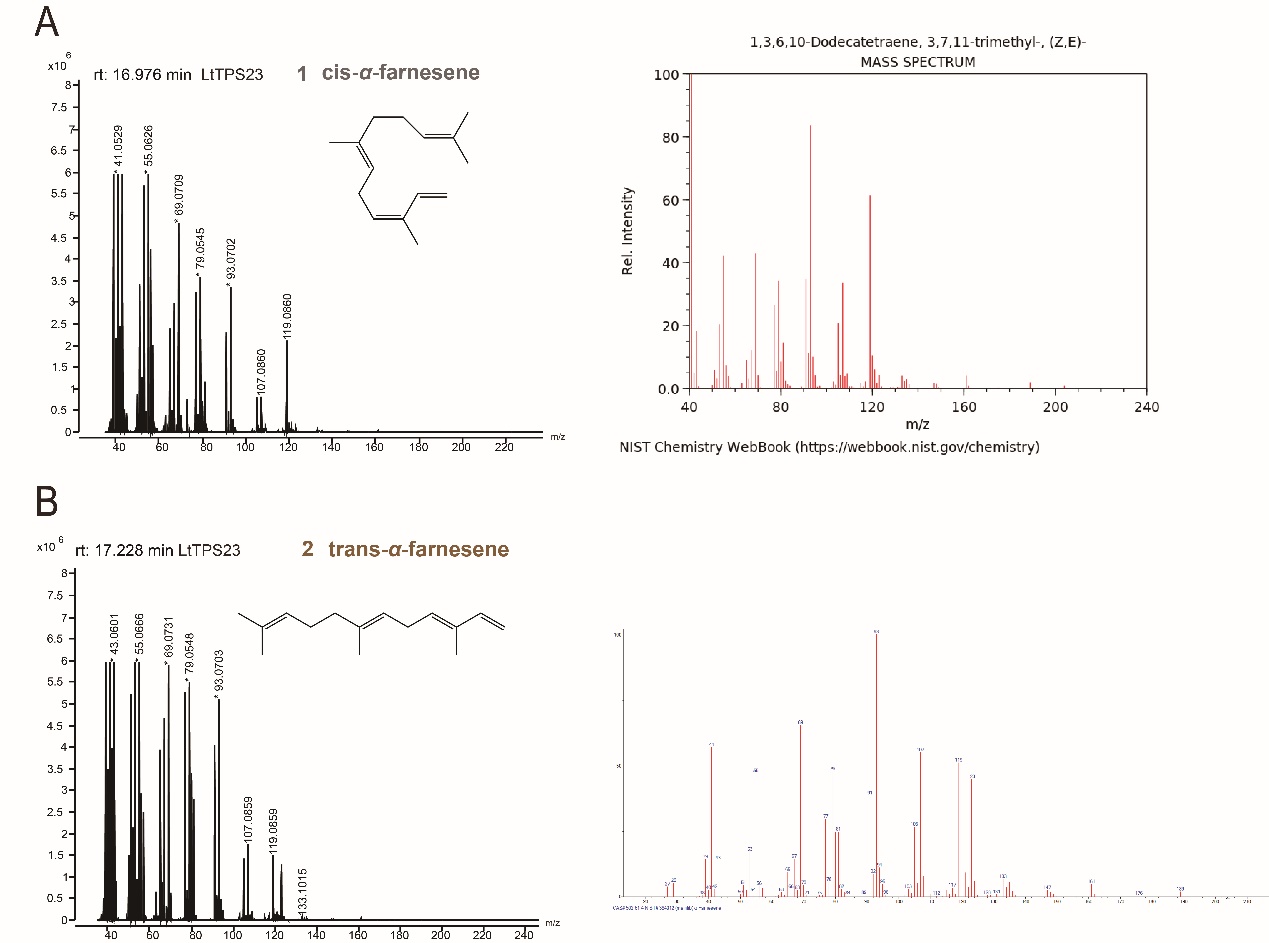


Figure S8. Characterization of the products of LtTPS23 with FPP. **A.** Mass spectrum of the product 1 from LtTPS23. Characterization of product 1 by NIST standard reference database. **B.** Mass spectrum of the product 2 from LtTPS23. Characterization of product 2 by Metabolomic mass spectrometry.


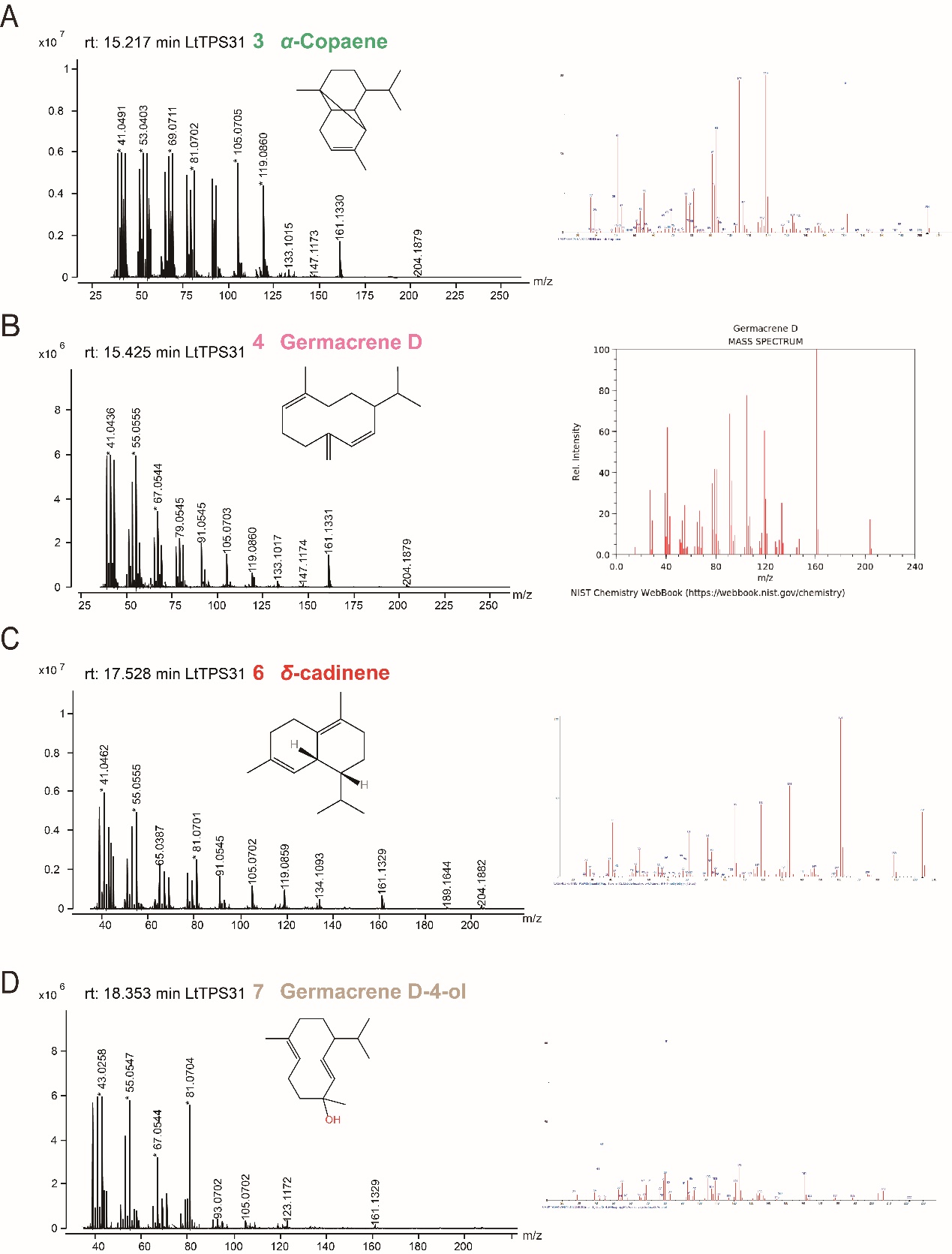


Figure S9. Characterization of the products of LtTPS31 with FPP. **A.** Mass spectrum of the product 3 from LtTPS31. **B.** Mass spectrum of the product 4 from LtTPS31. Characterization of product 4 by NIST standard reference database. **C.** Mass spectrum of product 6 from LtTPS31. **D.** Mass spectrum of the product 7 from LtTPS31. Characterization of product 3, 6 and 7 by Metabolomic mass spectrometry.


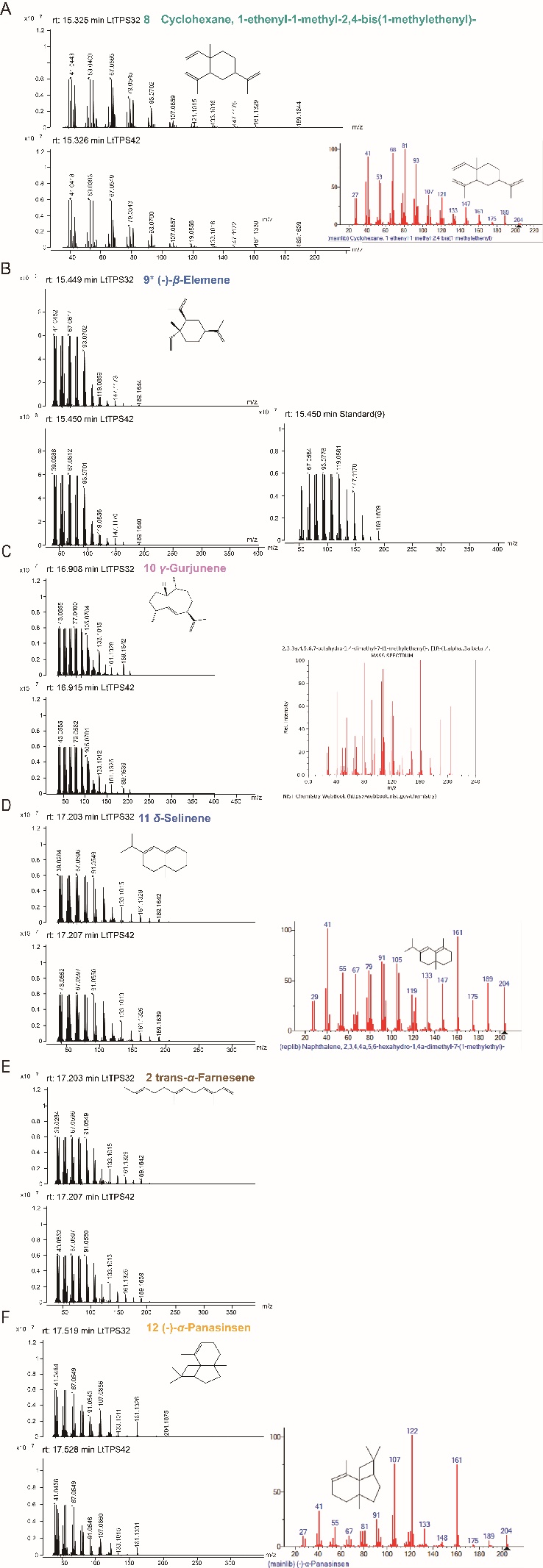


Figure S10. Characterization of the products of LtTPS32 and LtTPS42 with FPP. **A.** Mass spectrum of the product 8 from LtTPS32 and LtTPS42. **B.** Mass spectrum of the product 9 from LtTPS32 and LtTPS42. **C.** Mass spectrum of the product 10 from LtTPS32 and LtTPS42. **D.** Mass spectrum of the product 11 from LtTPS32 and LtTPS42. Characterization of product 11 by NIST standard reference database. **E.** Mass spectrum of product 2 from LtTPS32 and LtTPS42. **F.** Mass spectrum of the product 12. Characterization of the product 8, 10, 11 and 12 by NIST standard reference database.


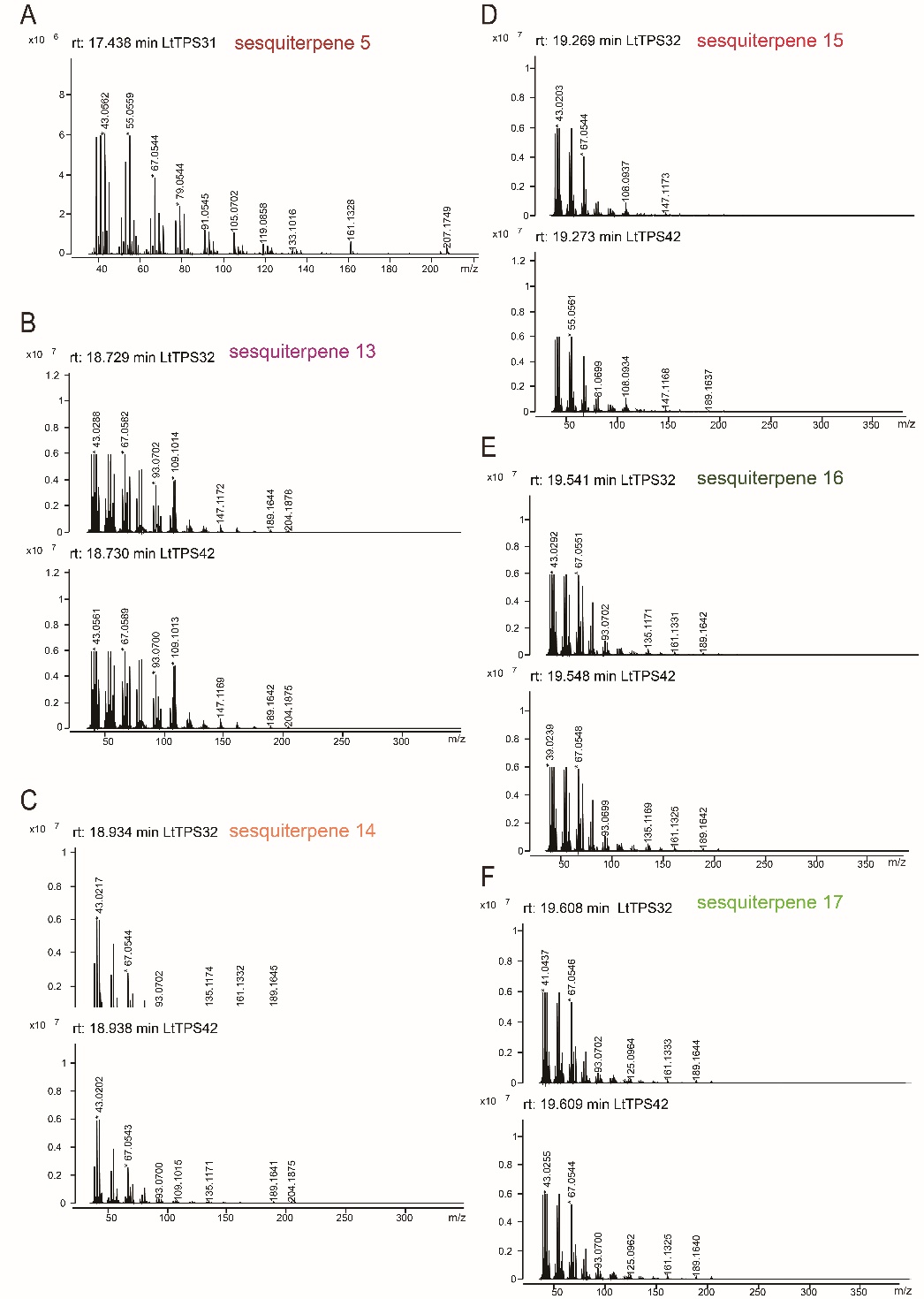


Figure S11. Characterization of the products of unkown sesquiterpenes products. **A.** Mass spectrum of the product 5 from LtTPS31. **B-F**. Mass spectrum of the product 13 to 17 from LtTPS32 and LtTPS42


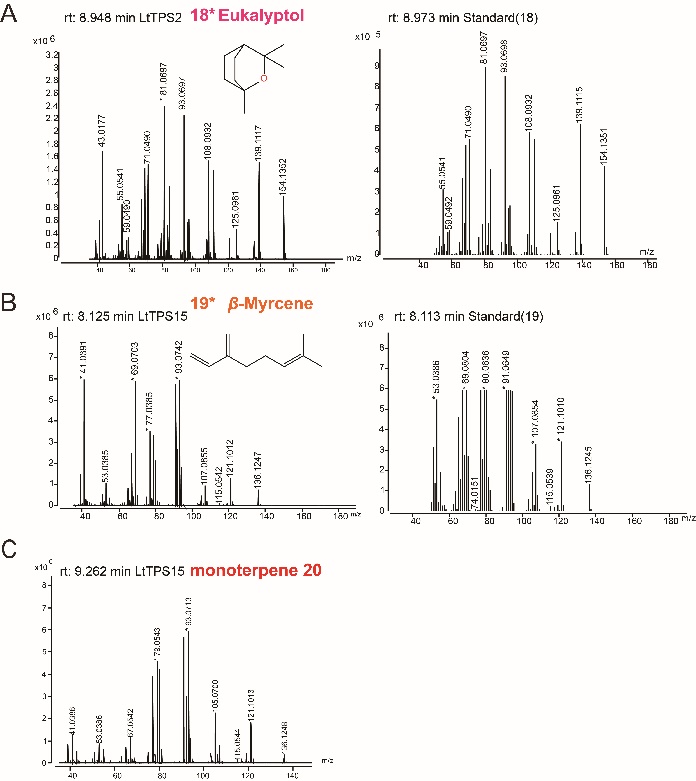


Figure S12. Characterization of the products of LtTPS2 and LtTPS15 with GPP. **A.** Mass spectrum of the product 18 from LtTPS2. **B.** Mass spectrum of the product 19 from LtTPS15. **C.** Mass spectrum of the product 20 from LtTPS15.

**
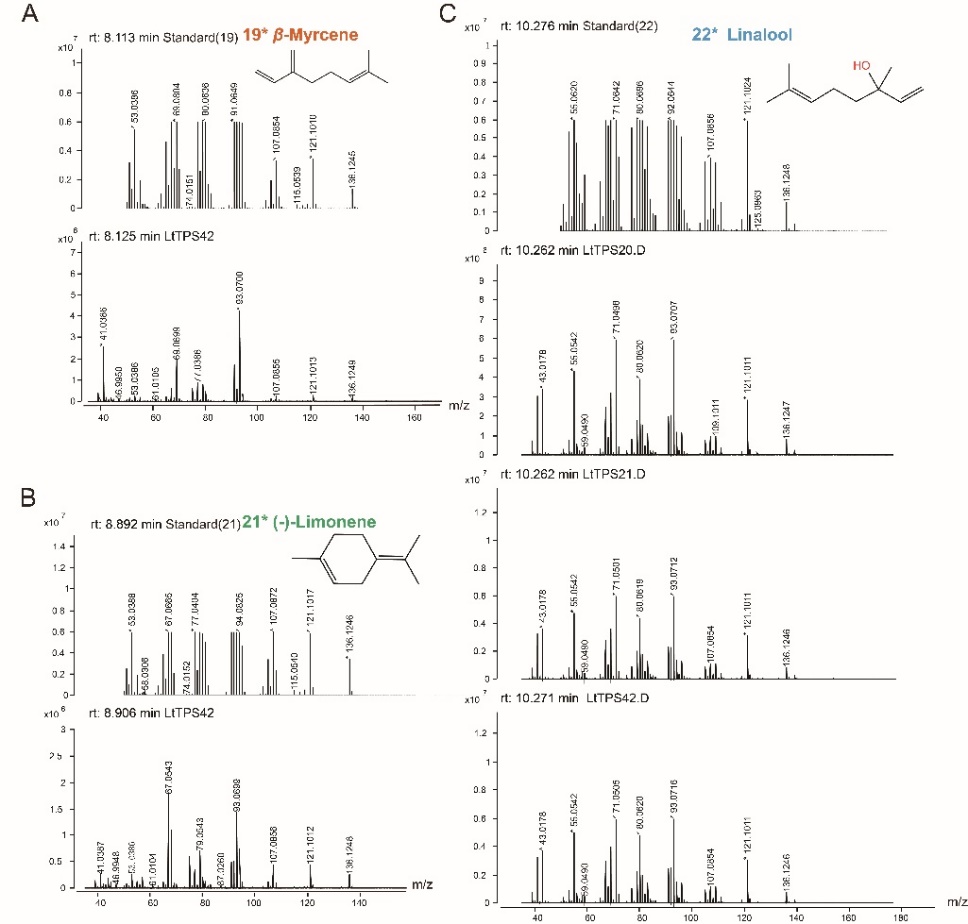
**

Figure S13. Characterization of the products of LtTPS20, LtTPS21 and LtTPS42 with GPP. **A.** Mass spectrum of the product 19 from LtTPS42. **B.** Mass spectrum of the product 21 from LtTPS42. **C.** Mass spectrum of the product 22 from LtTPS20, LtTPS21 and LtTPS45.

**
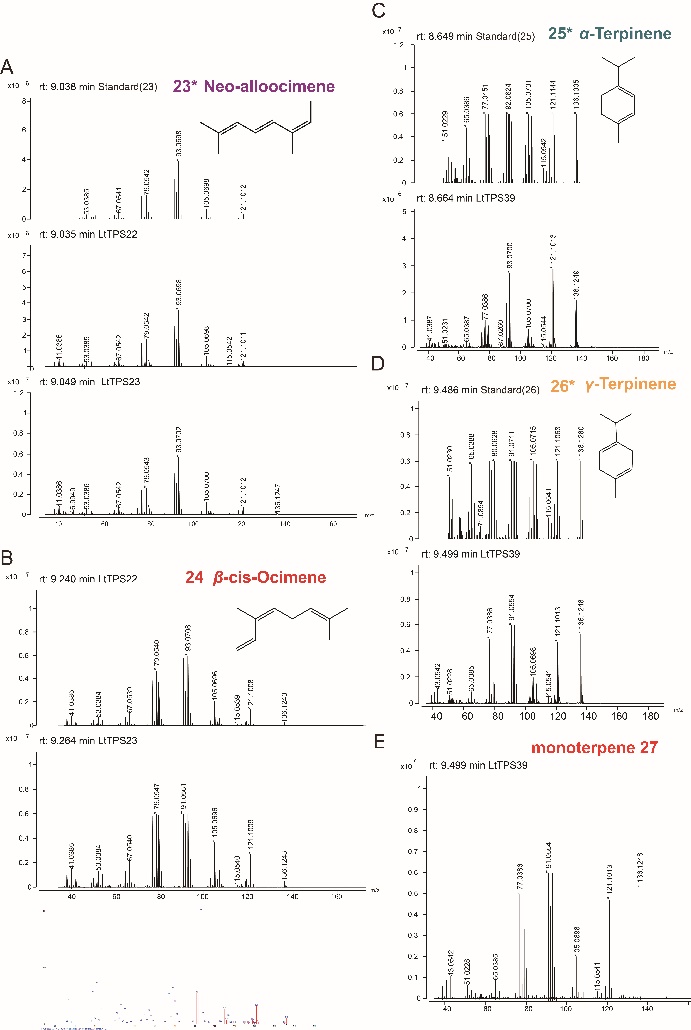
**

Figure S14. Characterization of the products of LtTPS22, LtTPS23 and LtTPS39 with GPP. **A.** Mass spectrum of the product 23 from LtTPS22 and LtTPS23. **B.** Mass spectrum of the product 24 from LtTPS22 and LtTPS23. Characterization of product 24 by Metabolomic mass spectrometry. **C.** Mass spectrum of the product 25 from LtTPS39. **D.** Mass spectrum of the product 26 from LtTPS39. E. Mass spectrum of the product 27 from LtTPS39.

Figure S15. Sequence alignment of LtTPS32 and LtTPS42.

Figure S16. Sequence alignment of LtTPS22 and LtTPS23.
